# Infrared optical signature reveals the source–dependency and along–transport evolution of dust mineralogy as shown by laboratory study

**DOI:** 10.1038/s41598-023-39336-7

**Published:** 2023-08-15

**Authors:** Claudia Di Biagio, Jean-François Doussin, Mathieu Cazaunau, Edouard Pangui, Juan Cuesta, Pasquale Sellitto, Milagros Ródenas, Paola Formenti

**Affiliations:** 1 Université Paris Cité and Univ Paris Est Creteil, CNRS, LISA, F-75013 Paris, France; 2 Univ Paris Est Creteil and Université Paris Cité, CNRS, LISA, F-94010 Créteil, France; 3 Istituto Nazionale di Geofisica e Vulcanologia, Osservartorio Etneo, Catania, Italy; 4 EUPHORE Labs., Fundación CEAM, 46980 Paterna, Valencia, Spain

**Keywords:** Atmospheric chemistry, Atmospheric chemistry, Climate and Earth system modelling, Environmental impact

## Abstract

Advancing knowledge of the mineralogical composition of dust is key for understanding and predicting its climate and environmental impacts. The variability of dust mineralogy from one source to another and its evolution during atmospheric transport is not measured at large scale. In this study we use laboratory measurements to demonstrate that the extinction signature of suspended dust aerosols in the 740 − 1250 cm^−1^ atmospheric window can be used to derive dust mineralogy in terms of the main infrared − active minerals, namely quartz, clays, feldspars and calcite. Various spectral signatures in dust extinction enable to distinguish between multiple global sources with changing composition, whereas modifications of the dust extinction spectra with time inform on size − dependent particles mineralogy changes during transport. The present study confirms that spectral and hyperspectral infrared remote sensing observations offer great potential for elucidating the size − segregated mineralogy of airborne dust at regional and global scales.

## Introduction

Mineral dust is amongst the most abundant and widespread aerosol specie on Earth^1–3^. Dust aerosol affects the climate system in multiple ways, including direct effects on atmospheric shortwave and longwave radiative budget^4–8^, indirect effect on liquid and ice cloud formation and properties^9–12^, contribution to biogeochemical cycles by acting as a source of nutrients for the ecosystems^13,14^, participation to atmospheric chemical reactions^15,16^, and contribution to air quality degradation and impacts on human health^17,18^. As demonstrated by extensive scientific work in the last decades, the strength and sign of these different effects depend on the dust mineralogical composition, i.e. the abundance, relative proportions and state of mixing of the different minerals composing the aerosols, which includes mainly silicates in the form of clays (kaolinite, illite, smectite, chlorite,..), quartz, and feldspars (orthose, albite,..), carbonates (calcite, dolomite), sulfates (gypsum) and iron and titanium oxides^19–21^. Indeed, different minerals show different features in terms of their spectral absorption and scattering properties, capacity to act as cloud condensation or ice nuclei particles, chemical reactivity, or solubility^22–28^. The knowledge of the dust mineralogical composition is fundamental for assessing its broad role in the Earth’s climate system and the environment.

Observations show that the mineralogy of dust aerosols is far from being homogeneous in the atmosphere^3,19,20,29–34^. First, due to the diverse soil mineralogy of the different source areas, the composition of dust varies with the region of emission, and this is both at the global, regional and local scales^19,35–37^. The dust mineralogical composition also changes with size: quartz, feldspars, and calcium − rich species are generally more abundant in the coarse mode component, while clays and iron oxides rely mostly on the fine fraction below 2 µm^20,38^. As a consequence of its size − dependence, the dust mineralogy modifies during transport because of the progressive loss of coarse particles due to gravitational settling^33,39,40^. Mixing of dust plumes with different origins and history may additionally occur in the atmosphere, further affecting the mineralogy of suspended dust.

As of today, available information on the dust mineralogical composition, its size − dependency and its spatio − temporal changes in the atmosphere remains still sparse and mostly limited to the specific conditions sampled during intensive field campaigns. The capability to get regional and global mapping of airborne dust mineralogy is still missing, a crucial limitation for properly developing and validating the representation of dust in Earth System Models, as well as for constraining its regional and global climate forcing^7,26,41^.

Due to their global coverage, frequency of observations, and multiyear duration, hyperspectral satellite measurements in various spectral ranges are currently used for characterizing desert dust abundance from daily to multi–year scale, in terms of horizontal and vertical distributions (for example^42–46^). Because the different minerals composing the fine and coarse fractions of dust show different spectral absorption signatures, these satellite observations can also be potentially used to detect the presence of diverse minerals and reconstruct their relative proportions in the dust aerosols^28,47,48^. Therefore, they offer the potential to provide highly valuable knowledge on size − resolved dust mineralogy and its spatio − temporal pattern. Ultraviolet − visible (UV − Vis, ~300 − 800 nm) and Visible to Short Wavelength Infrared (VSWIR, ~350 − 2500 nm) regions contain the absorption signatures of iron oxides (hematite, goethite), clays and carbonates, more abundant in the dust fine − fraction, whereas the longwave infrared region (LWIR, ~2.5 − 25 µm) is more sensitive to the presence of coarse − sized silicates and carbonates characterized by specific lattice vibrational–rotational transitions in this spectral region, including quartz and feldspars to which VSWIR radiation is not sensitive^23,28,48,49^.

Up to now, efforts aimed at quantitatively exploiting satellite observations to derive dust mineralogy from their optical signature have focused on the UV − Vis and VSWIR regions of the spectrum, therefore mostly looking at dust small − sized minerals^50–52^. Using the Deep Space Climate Observatory (DSCOVR) Earth Polychromatic Imaging Camera (EPIC), Go et al.^50^ developed a retrieval algorithm to determine the total iron − oxide mass fractions in dust and its speciation in hematite and goethite minerals. Sanwlani and Das^52^ use MODIS (Moderate Resolution Imaging Spectroradiometer) spectral optical depth observations at discrete wavelengths to reconstruct the main mineralogical features of the detected dust plumes. The EMIT mission (Earth Surface Mineral Dust Source Investigation)^51^, which integrated the International Space Station (ISS) in June 2022, will employ VSWIR reflectance observations to retrieve new data on the mineralogy of source soils (clays, iron oxides, sulfates, carbonates) from dust − emitting regions worldwide.

Hyperspectral observations in the LWIR, as those performed by IASI (Infrared Atmospheric Sounding Interferometer)^53^ and the IASI − NG (Next Generation)^54^ instruments, can complement information obtained from UV − Vis and VSWIR sensors by detecting coarse − sized mineral components, including silicates, in particular quartz, clays, and feldspars, and carbonates. In the infrared atmospheric window region located at 8–13.5 µm (740–1250 cm^–1^, i.e. the spectral region of the infrared spectrum with relatively low absorption by atmospheric gases) the LWIR dust signature arises from the overlapping contribution of quartz, clays and feldspars above 900 cm^−1^, and separate bands for calcite (876 cm^−1^) and quartz (800 and 777 cm^−1^) below 900 cm^−1^^23,28^. Past literature based on laboratory transmission, reflectance, and emission measurements on pellet, thin film and soil samples has demonstrated that the LWIR spectra of mixtures of minerals in this spectral region can be reconstructed as the linear combination of the spectra of its single components', an approach named linear spectral mixing (LSM)^48,55–57^, which is used to investigate the surface composition of the Earth and Mars^58,59^. The LSM analysis of LWIR spectra of natural dust mixtures can be used to retrieve quantitatively its mineralogical composition. A first successful attempt of application of an LSM–like method to atmospheric IASI thermal infrared spectra has been conducted for retrieving information on the coarse dust composition for a case study over the Gobi desert^60^.

In the present study we explore the potential of LWIR spectral extinction signal of airborne mineral dust to inform about its mineralogy at the global − scale level. We use data of LWIR high − resolution extinction spectra, mineralogical composition and size distribution obtained from laboratory chamber experiments on suspended natural dust aerosols from nineteen source regions worldwide with contrasting mineralogy^61^. The dust aerosols, generated with a system that mimics the saltation and sandblasting of soils during wind erosion, are injected in the CESAM large simulation chamber^62^ and aged for a time between 60 and 120 min under the effect of gravitational settling. The progressive loss of coarse particles during aging time allows to reproduce the temporal changes of dust size distribution and mass load as observed in the real atmosphere from emission to medium − to long − range transport^61,63,64^, comparable to what is observed for African dust from source areas towards the Mediterranean and the Atlantic^20,32,39,40,65–67^. The LSM analysis is applied to the dust extinction spectra measured during the experiments. A library of reference extinction spectra for single minerals composing dust including silicates (phyllosilicates (mica, serpentine/kaolinite, smectite, chlorite, and talc/pyrophyllite groups) and tectosilicates (feldspar and silica groups) families), carbonates, diatomite, and sulfates, constructed from data available in the literature for suspended particles and compressed pellets is used in the analysis (see Methods).

We take advantage of this unique dataset to test the capability of the LSM approach to provide consistent information on dust aerosol mineralogical composition, its size − and source − dependency, and its modifications due to size − selective coarse depletion. The bulk mineralogy and mass concentration derived from X‒ray diffraction (XRD) and X‒ray fluorescence (XRF) on dust particles collected on filters over the whole duration of each experiment is compared to the retrieved mineralogical composition and reconstructed mass concentration of the suspended dust from LSM analysis. Thanks to the specificity of the spectral features in the 740 to 1250 cm^−1^ (8–13.5 µm) range, it is possible to reproduce the high − resolution LWIR extinction spectra of natural dust mixtures starting from a limited number of single mineral references. A quantitative estimate of the relative amounts of phyllosilicates, quartz, feldspars, carbonates, as well as minor components as sulfates or diatomite, can be derived in agreement with XRD analysis and literature observations. Based on their unique extinction signature, the mineralogical composition of samples of dust from different sources can be retrieved. Modifications of the dust extinction spectra with residence time in the chamber are shown to inform on modifications in clays partitioning and variations in feldspars content with evolving size distribution. Realistic estimation of the total dust mass concentration and its temporal evolution is provided by LSM. The analysis performed in the present study demonstrates the possibility to retrieve consistent mineralogical signatures and mineral quantification from the measurements of high − resolution extinction spectra of dust in the LWIR spectral window and to follow the evolution of mineralogy with size − selective gravitational settling as occurring during large scale dust transport. These results support new ways of exploiting existing and future satellite measurements and suggest that LWIR remote sensing spectroscopy can help in significantly advance our knowledge of dust mineralogy at a global scale by mapping diversity of origin and properties.

## Results

### LSM fitting of experimental dust spectra

**Figure 1:**
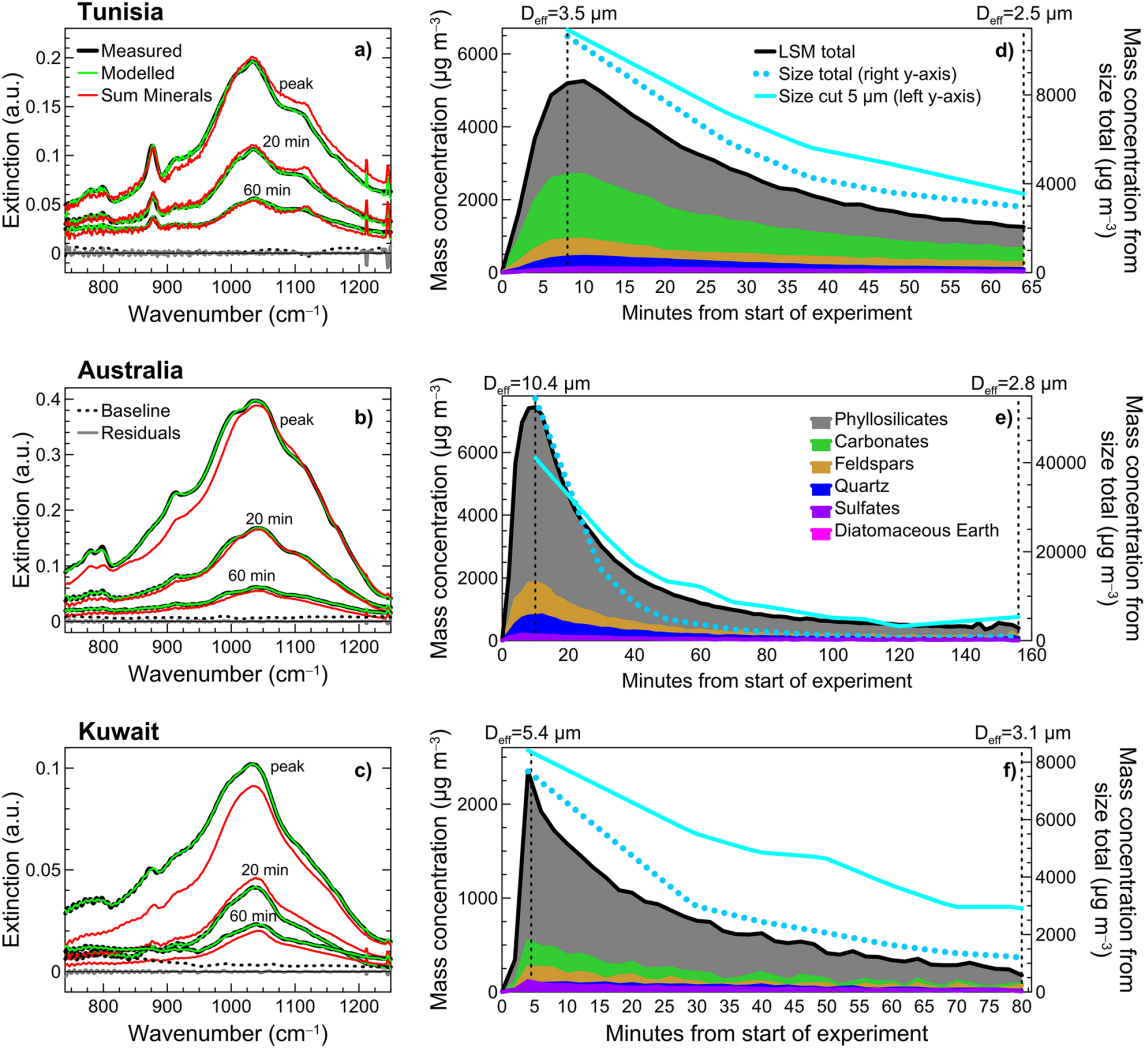
Illustration of Linear Spectral Mixing (LSM) analysis and comparison to online measurements. (**a**, **b**, **c**) Measured and modelled extinction spectra (expressed as absorbance in log10 convention) for (**a**) Tunisia, (**b**) Australia, and (**c**) Kuwait samples at the peak of the dust injection in the CESAM chamber and after 20 and 60 min aging time. The modelled spectra from LSM analysis is the sum of the single minerals contributions (Sum Minerals, plotted in red) and the baseline offset term (Baseline, plotted in black dotted) (see Methods). The Residual (difference between measured and modelled signal) is also shown (plotted in grey). Examples of baseline and residuals are reported at the bottom of each plot in reference only to the spectra at 60 min. The horizontal black line represents the 0 value. (**d**, **e**, **f**) Temporal evolution of the total mass concentration of dust aerosols and minerals contributions obtained from LSM modelling and comparison with the total mass concentration of dust in CESAM obtained from online size distribution data. Mass concentrations obtained both considering full size distribution (size total) and size cut at 5 µm diameter, i.e. integrating only diameters below 5 µm, (size cut 5 µm) are reported. The size total data are plotted against the right y − axis while the size cut 5 µm data are plotted against the left y − axis. The effective diameter (D_eff_) calculated from online size distribution data at the beginning and at the end of the experiments is also indicated. Note that the peak of dust injection is reached more rapidly for Kuwait sample (panel **f**) compared to Tunisia and Australia (panels **d**–**e**) which causes a sharper increase of the mass concentration curve at the initial stages of the experiment.

Results of LSM analysis applied to chamber experimental data are illustrated (Fig. 1) for three experiments with dust aerosols from Tunisia, Australia, and Kuwait, showing different extinction intensities and spectral features. The comparison between measured and modelled spectra, as taken at the peak of the dust injection in CESAM and after 20 and 60 min of aging is reported for the three samples (Fig. 1a–c). The spectral extinction versus LSM fitting is also illustrated for all the remaining sixteen samples in this study (Supplementary Fig. 1). As seen in Fig. 1a–c, the difference between measured and modelled spectra (residual) is low, meaning that the model provides a very good fit of the measured spectra at the different times of the experiment. The Normalized Root Mean Square Error is low, typically between 1 and 5%. The modelled spectra (which is given by the sum of minerals contribution, i.e. the portion of the extinction spectrum reconstructed as sum of single mineral extinctions, plus a baseline offset term) very well reproduce band positions and intensities. The minerals contribution (in red in Fig. 1a–c) accounts for most of the modelled signal and reproduces the different absorption bands in the spectra. The polynomial baseline (in dotted black in Fig. 1a–c), which can assume both positive and negative values, shows a smoothed spectral variation and contributes to a fraction of the modelled signal that is usually less than 20% at 1000 cm^−1^ but increases up to 50% towards low wavenumbers.

### Minerals abundance retrieved from LSM analysis

The LSM procedure allows to resolve both overlapping and distinct mineral absorbing features and the contribution of different species to dust mass concentration is reconstructed as a function of time. Results are illustrated for samples from Tunisia, Australia and Kuwait (Fig. 1d–f; reporting absolute mass concentrations), and summarized in Table 1 (as percent mineral contributions) for the nineteen dust samples of this study. Globally, realistic mineral abundances are simulated, and sample − to − sample differences are identified, in overall agreement with literature–based knowledge.

**Table 1:** Mineralogy for fresh and aged dust samples retrieved from linear spectral mixing (LSM) analysis. Mineralogy retrieved for each sample at the peak of injection in the CESAM simulation chamber (labelled as fresh) and at the end of the experiment (labelled as aged). Percent mass fraction for each mineral is derived by using as total mass concentration the one derived from LSM analysis. For aged conditions the number of minutes of aging in the chamber is also indicated in parenthesis in the first column. Fresh and aged values are the average (± standard deviation) of five consecutive spectra. The total phyllosilicates mass includes the contribution of illite, kaolinite, montmorillonite, chlorite, chamosite, serpentine and talc. The illite, kaolinite and montmoriilonite contributions to phyllosilicates are reported in the table. The total feldspars includes albite, oligoclase and orthoclase. The effective diameter D_eff_ calculated from online size distribution measurements during experiments is also reported. Its uncertainty is estimated at 25%^61,63^.

Sample name	Phyllosilicates (%)				Quartz (%)	Feldspars (%)	Calcite (%)	Dolomite (%)	Anhydrite (%)	Diatomaceous Earth (%)	D_eff_ (µm)
	Total	Illite (%)	Kaolinite (%)	Montmorillonite (%)							
Tunisia − fresh	46 ± 1	34 ± 2	1 ± 1	9 ± 2	5 ± 1	10 ± 1	35 ± 1	0 ± 0	2 ± 0	0 ± 0	3.5 ± 0.9
Tunisia − aged (62)	41 ± 2	24 ± 3	0 ± 0	13 ± 1	5 ± 0	13 ± 1	31 ± 1	0 ± 0	3 ± 0	0 ± 0	2.5 ± 0.6
Morocco − fresh	48 ± 0	34 ± 0	5 ± 1	10 ± 1	5 ± 0	2 ± 1	41 ± 1	0 ± 0	4 ± 0	0 ± 0	4.5 ± 1.1
Morocco − aged (100)	48 ± 3	36 ± 4	11 ± 2	2 ± 1	4 ± 1	1 ± 1	39 ± 4	0 ± 0	8 ± 1	0 ± 0	2.6 ± 0.7
Libya − fresh	83 ± 1	43 ± 4	17 ± 4	24 ± 7	2 ± 0	6 ± 1	6 ± 1	0 ± 0	3 ± 0	0 ± 0	9.5 ± 2.4
Libya − aged (184)	84 ± 5	34 ± 8	49 ± 5	1 ± 1	2 ± 1	6 ± 6	4 ± 3	0 ± 0	4 ± 2	0 ± 0	1.9 ± 0.5
Algeria − fresh	82 ± 1	27 ± 2	5 ± 1	50 ± 3	4 ± 0	12 ± 1	1 ± 0	0 ± 0	1 ± 0	0 ± 0	8.5 ± 2.1
Algeria − aged (120)	82 ± 3	41 ± 8	19 ± 2	22 ± 7	6 ± 2	10 ± 3	0 ± 0	1 ± 1	3 ± 1	0 ± 0	3.0 ± 0.8
Mauritania − fresh	81 ± 2	23 ± 4	0 ± 0	58 ± 6	3 ± 1	5 ± 2	11 ± 0	0 ± 0	0 ± 0	0 ± 0	7.0 ± 1.8
Mauritania − aged (82)	88 ± 4	32 ± 5	0 ± 0	56 ± 5	8 ± 2	2 ± 3	2 ± 3	0 ± 0	0 ± 0	0 ± 0	2.8 ± 0.7
Niger − fresh	56 ± 2	43 ± 2	12 ± 1	1 ± 1	22 ± 1	21 ± 3	0 ± 0	0 ± 0	2 ± 0	0 ± 0	5.1 ± 1.3
Niger − aged (138)	67 ± 4	27 ± 3	40 ± 1	0 ± 0	22 ± 2	11 ± 6	0 ± 0	0 ± 0	1 ± 1	0 ± 0	1.1 ± 0.3
Mali − fresh	75 ± 3	33 ± 4	7 ± 2	35 ± 6	7 ± 2	14 ± 3	0 ± 0	0 ± 0	5 ± 1	0 ± 0	4.1 ± 1.0
Mali − aged (56)	80 ± 10	36 ± 9	16 ± 4	27 ± 9	11 ± 5	3 ± 6	0 ± 0	0 ± 0	7 ± 1	0 ± 0	3.3 ± 0.8
Bodélé − fresh	44 ± 8	31 ± 4	4 ± 3	7 ± 4	17 ± 3	26 ± 4	0 ± 0	0 ± 0	8 ± 2	6 ± 1	8.4 ± 2.1
Bodélé − aged (138)	57 ± 4	12 ± 6	27 ± 4	9 ± 5	19 ± 12	12 ± 3	4 ± 4	0 ± 0	0 ± 0	8 ± 5	3.3 ± 0.8
Ethiopia − fresh	76 ± 1	28 ± 2	0 ± 0	48 ± 2	9 ± 0	9 ± 1	4 ± 1	0 ± 0	0 ± 0	2 ± 0	8.4 ± 2.1
Ethiopia − aged (132)	75 ± 9	22 ± 9	0 ± 0	53 ± 6	6 ± 5	7 ± 10	9 ± 2	0 ± 0	0 ± 0	4 ± 1	3.3 ± 0.8
Saudi Arabia − fresh	56 ± 1	41 ± 2	3 ± 0	12 ± 3	1 ± 0	9 ± 1	33 ± 0	0 ± 0	0 ± 0	0 ± 0	8.5 ± 2.1
Saudi Arabia − aged (132)	44 ± 1	33 ± 3	8 ± 2	3 ± 1	0 ± 0	9 ± 1	44 ± 1	0 ± 0	0 ± 0	0 ± 0	3.3 ± 0.8
Kuwait − fresh	75 ± 2	34 ± 5	0 ± 0	40 ± 7	1 ± 1	8 ± 2	13 ± 1	0 ± 0	5 ± 0	0 ± 0	5.4 ± 1.4
Kuwait − aged (60)	70 ± 7	42 ± 9	0 ± 0	28 ± 5	3 ± 2	9 ± 6	11 ± 3	0 ± 0	6 ± 1	0 ± 0	3.1 ± 0.8
Gobi − fresh	54 ± 1	46 ± 2	0 ± 0	7 ± 1	15 ± 0	13 ± 2	12 ± 0	0 ± 0	6 ± 0	0 ± 0	3.1 ± 0.8
Gobi − aged (78)	61 ± 1	52 ± 1	0 ± 0	8 ± 1	15 ± 0	2 ± 1	14 ± 0	0 ± 0	9 ± 0	0 ± 0	1.5 ± 0.4
Taklimakan − fresh	65 ± 1	42 ± 1	0 ± 0	0 ± 0	4 ± 0	15 ± 1	13 ± 2	0 ± 0	3 ± 0	0 ± 0	7.3 ± 1.8
Taklimakan − aged (124)	72 ± 4	64 ± 5	0 ± 0	0 ± 0	3 ± 0	5 ± 4	16 ± 2	0 ± 0	4 ± 1	0 ± 0	2.8 ± 0.7
Arizona − fresh	72 ± 1	27 ± 8	0 ± 0	30 ± 4	4 ± 1	12 ± 0	7 ± 1	0 ± 0	4 ± 0	0 ± 0	8.5 ± 2.1
Arizona − aged (72)	70 ± 4	46 ± 7	0 ± 0	19 ± 4	3 ± 1	16 ± 8	7 ± 3	0 ± 0	4 ± 2	0 ± 0	3.9 ± 1.0
Atacama − fresh	57 ± 1	29 ± 5	0 ± 0	28 ± 6	1 ± 0	7 ± 1	34 ± 1	0 ± 0	2 ± 0	0 ± 0	6.9 ± 1.7
Atacama − aged (128)	59 ± 3	49 ± 4	5 ± 1	5 ± 2	2 ± 1	5 ± 2	32 ± 3	0 ± 0	3 ± 1	0 ± 0	2.7 ± 0.7
Patagonia − fresh	55 ± 1	16 ± 3	0 ± 0	39 ± 2	20 ± 1	17 ± 1	0 ± 0	1 ± 0	8 ± 1	0 ± 0	10.9 ± 2.7
Patagonia − aged (64)	59 ± 1	14 ± 5	0 ± 0	45 ± 4	23 ± 1	11 ± 2	0 ± 0	0 ± 0	7 ± 1	0 ± 0	3.6 ± 0.9
Namib-1 − fresh	36 ± 0	23 ± 1	0 ± 0	13 ± 1	0 ± 0	10 ± 0	46 ± 1	0 ± 0	8 ± 0	0 ± 0	9.4 ± 2.4
Namib-1 − aged (82)	36 ± 2	25 ± 2	0 ± 0	10 ± 1	0 ± 0	11 ± 2	43 ± 1	0 ± 0	11 ± 0	0 ± 0	4.0 ± 1.0
Namib-2 − fresh	91 ± 0	9 ± 2	0 ± 0	82 ± 2	0 ± 0	9 ± 0	0 ± 0	0 ± 0	0 ± 0	0 ± 0	9.4 ± 2.4
Namib-2 − aged (124)	84 ± 5	8 ± 5	0 ± 0	77 ± 2	0 ± 0	16 ± 5	0 ± 0	0 ± 0	0 ± 0	0 ± 0	1.9 ± 0.5
Australia − fresh	76 ± 1	12 ± 4	3 ± 1	62 ± 5	8 ± 1	14 ± 1	0 ± 0	0 ± 0	2 ± 0	0 ± 0	10.4 ± 2.6
Australia − aged (122)	76 ± 6	23 ± 5	0 ± 0	54 ± 3	6 ± 1	12 ± 8	0 ± 0	0 ± 0	6 ± 2	0 ± 0	2.8 ± 0.7

Phyllosilicates represent between 36 and 91% in mass for all samples at the peak of injection in CESAM, values which are consistent with field investigations of African and Asian dusts^19,20,31,68^. Illite dominates the clay speciation for North African and Chinese samples, in line with observations^29,30,36^, whereas montmorillonite is dominant for Mauritania, Ethiopia, Kuwait, Patagonia, Namib − 2 and Australia samples. Kaolinite contributes significantly to clay speciation for Northern African and Sahelian samples, but it is almost absent in all the other regions. Quartz is lower than 5% for most samples but reaches up to 21% content for Sahelian, Gobi, Patagonia and Australia samples at injection peak. Quartz − rich samples in Sahel and Asia are also reported in the literature^19,30,31^. Feldspars are present in variable amounts ranging between 1 and 26% in all samples, with the highest values for Niger and Bodélé. The geographical variability in dust feldspars content is in agreement with soil composition showing the highest feldspars content in the Sahel^69^. However, available field observations have shown a much lower contribution (< 2%) of feldspars from Niger and Bodélé sources^19^. Calcite is found to be the highest in Tunisia, Morocco, Saudi Arabia, Atacama and Namib − 1 samples (33 to 46% at the peak of injection in CESAM) and less than 13% for the other samples, with exception of Niger, Mali, Patagonia, Namib − 2 and Australia for which no calcite is detected. Geographical distribution of calcite between analyzed samples well reflects the global distribution of this mineral in soil samples^69^ and field aerosol observations^20,29–31^. Dolomite has a negligible contribution to reconstructed mineralogy, in line with previous mineralogical analysis in Northern Africa^19,20^, while anhydrite is between 0 and 8% for all samples at the peak of injection. Field observations of sulfates are limited to crystalline gypsum, shown to contribute a few percent to the dust mass^19,20^. Diatomaceous Earth has no contribution to all samples, with the exception of Bodélé (6% at peak) and a small contribution to Ethiopia (2%). The Bodélé depression is a diatomite − rich area, and aerosols from this zone contain resuspension of detrital diatoms^35^. We are capable therefore to identify this unique feature of Bodélé features from our dataset and analysis.

### Temporal evolution of the mineralogical composition from LSM analysis

Results reported in Fig. 1d–f and Table 1 illustrate the evolution of LSM reconstructed mineralogy with a lifetime in the chamber up to more than 2 h aging that corresponds to a gravitational processing equivalent to that potentially occurring over medium − to long − range transport. The effective diameter (D_eff_) of dust, also reported in Fig. 1d–f and Table 1, varies in the range 3.1–10.9 µm for fresh conditions (injection peak) to 1.1 − 4.0 µm for aged dust, values which reproduce the size − selective changes observed in the field within 1 to 5 days of transport^39,65,66,70^. Results indicate that the dust mineralogy retrieved from LSM changes from the beginning to the end of each experiment. However, no systematic tendencies are identified for all samples. The content of phyllosilicates, calcite, quartz and feldspars is alternatively increasing or decreasing for the various samples for fresh and aged conditions. A closer look at the data however evidences some consistent features. In terms of phyllosilicates speciation, it can be noticed that when kaolinite is present, it tends to increase systematically from fresh to aged conditions, with a maximum of 32% increase for Libya (that is the % contribution of kaolinite to total dust mass goes from 17 to 49% from fresh to aged conditions). As a consequence, kaolinite, which is never the dominant clay specie for fresh dust as discussed in the previous paragraph, becomes the dominant clay specie for aged Libya, Niger and Bodélé dust, in good agreement with field observations^19^. This could potentially suggest that the fresh dust in CESAM is representative of the first stages of dust emissions in atmosphere, a condition which has been rarely sampled in the field^20,71^. Montmorillonite shows on the contrary a tendency to decrease (down to 30% variation) going from fresh to aged conditions, whereas illite increases with aging for most samples and almost systematically when kaolinite is not present. While the speciation between different clay species seems to be affected by gravitational settling, the total amount of phyllosilicates is observed to either remain almost constant with time (8 cases), or to slightly decrease (4 cases) or increase (7 cases) showing however maximum variation within 12%. One consistent feature observed is that the increase/decrease of phyllosilicates is opposite to feldspars. On the contrary, quartz content remains mostly unchanged between fresh and aged conditions with variations lower than 5% which do not co–occur with those of phyllosilicates or other minerals. Calcite behaves similarly, with variations going up to 10% in both directions and not clearly correlated to other mineral changes.

### Comparison of mineralogy retrieved from LSM and measured by X–ray diffraction (XRD)

**Figure 2:**
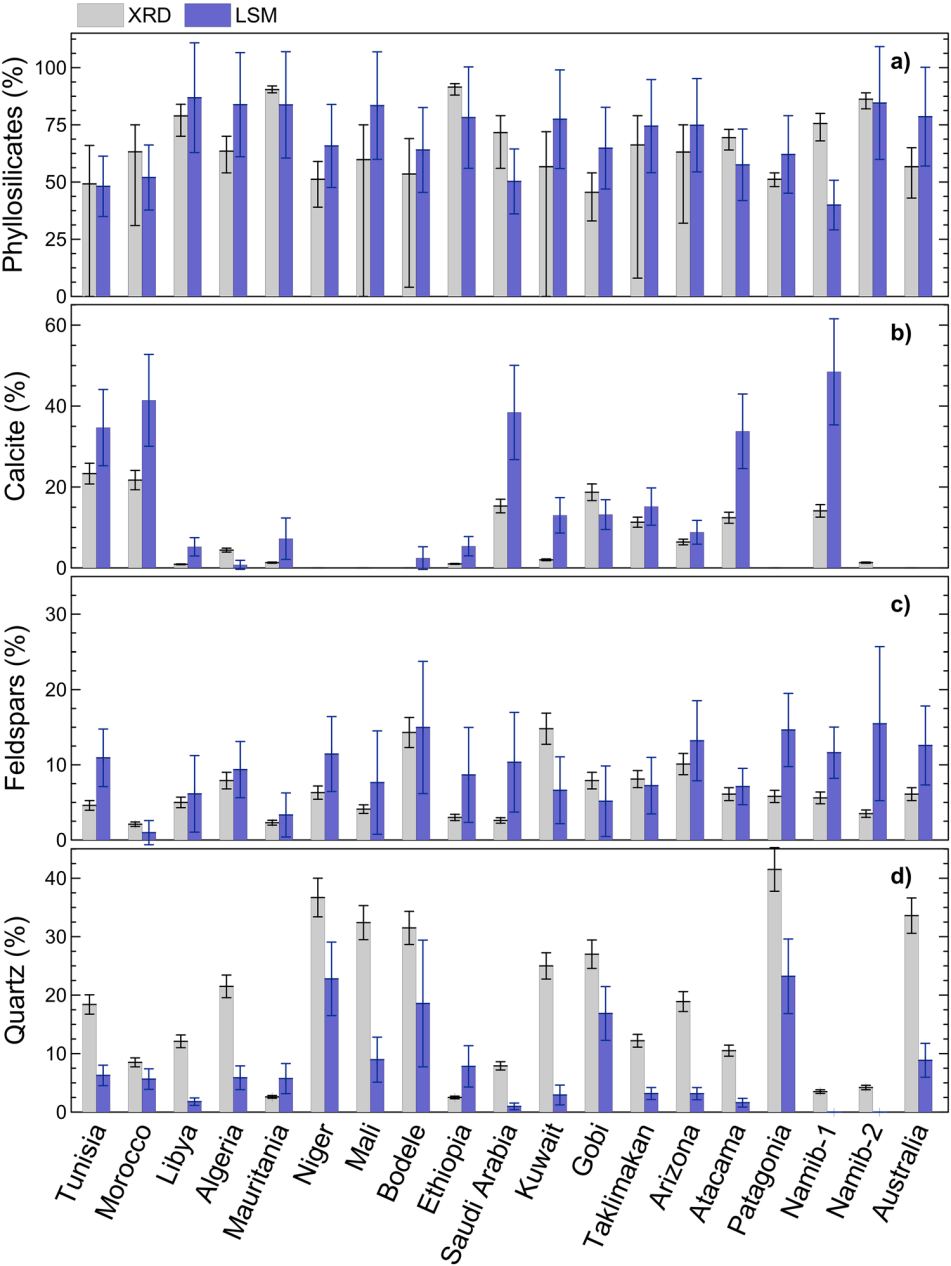
Comparison of mineralogy retrieved from LSM and measured by X–Ray Diffraction (XRD). Data represent the comparison (percent content in mass) for (**a**) phyllosilicates, (**b**) calcite, (**c**) feldspars, and (**d**) quartz. The XRD data are measured on dust samples collected on filters and represent mineralogy integrated over the duration of each experiment^61^. Uncertainty on XRD data is obtained from XRD single mineral calibration and total mass estimation^61^. For comparison purposes, and since only the crystalline phase is visible to XRD, the % content of minerals from LSM is rescaled removing from the total mass the contribution of non − crystalline minerals such as anhydrite and diatomaceous earth, and also that of ammonium sulfate. The LSM data are averaged over the same interval of XRD filter sampling. The uncertainty on LSM average values is calculated as the quadratic combination of mass concentration uncertainty (± 27%, obtained with the error propagation formula considering ± 19% uncertainty on both single mineral and total dust mass from LSM analysis) and the standard deviation over the average period.

The comparison between LSM and XRD derived experiment–averaged mineralogy for the nineteen analyzed samples in this study (Fig. 2 and Supplementary Fig. 3) confirms that the main features of dust mineralogy are well reproduced by LSM. Phyllosilicates are in good agreement, both in terms of absolute values (in the range 40 − 90% content) and sample − to − sample variability when comparing the two techniques. For calcite and feldspars, the LSM also well reproduces the sample − to − sample variability as obtained in XRD, but tends to overestimate the absolute content of these minerals. Calcite from LSM analysis, in particular, shows the highest values for Northern African (Tunisia, Morocco), Saudi Arabia, Atacama, and Namib − 1 samples. Quartz is identified in almost all samples, in line with XRD observations, with the exception of Namib − 1 and Namib − 2 samples for which no quartz is retrieved from LSM. These samples show amongst the lowest quartz content from XRD analyses as well. Contrarily to calcite and feldspars, quartz is significantly underestimated in LSM compared to XRD, with the only exception of Mauritania and Ethiopia.

### Comparison of total mass concentration retrieved from LSM and measured by size distribution and X–ray fluorescence (XRF)

The total mass of dust aerosols reconstructed from LSM (sum up of the minerals contributions; Fig. 1d–f) increases as the starting of the dust injection in CESAM, maximizes the peak of injection (reaching up to tenths of mg m^−3^) and reduces with residence time in the chamber (down to values less than 1 mg m^−3^ after 120 min aging), following the intensity changes in measured spectra. The lowest detected mass concentration for single minerals is around 5 µg m^–3^, a value which can be assumed as detection limit in the present analysis. The temporal evolution of the total mass concentration from LSM compares well with the behavior derived from size distribution measurements from particle sizers connected online to the chamber (Fig. 1d–f), suggesting that the dynamic of the dust population is properly captured by LSM inversion. Nevertheless, the absolute value is up to a factor 10 lower that the total mass from size distribution data (as observed for all samples). The size/LSM mass ratio is related to the fraction of coarse particles in the dust and decreases for reducing the coarse component with aging in the chamber. It should be noted that the mass concentration from size distribution is highly uncertain because of the low concentrations of coarse particles and size − selective particle losses along sampling tubes^61^. Cutting the size distribution at 5 µm in diameter (Fig. 1d–f) would significantly improve the comparison, suggesting that the infrared observations are more sensitive to particles in this size range.

**Figure 3:**
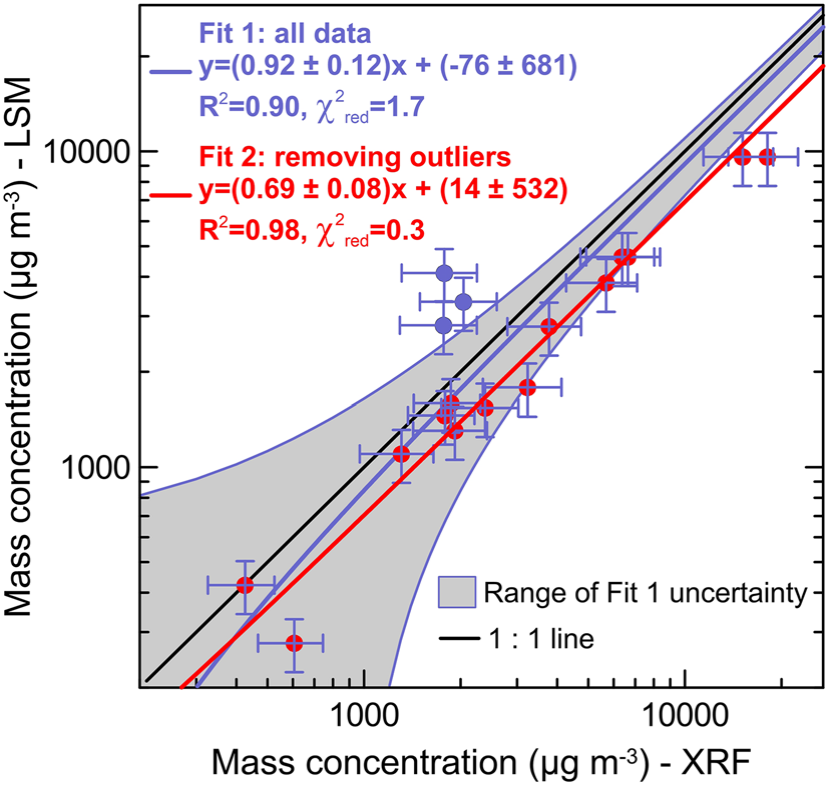
Comparison of mass concentration retrieved from LSM and measured by X–Ray Fluorescence (XRF). The XRF data are measured on dust samples collected on filters and represent mass concentration integrated over the duration of each experiment^64^. The LSM data are averaged over the same interval of XRF filter sampling, and the XRF concentrations are corrected for particle losses along sampling lines^61,64^. Data points in blue evidence three outliers in the dataset, corresponding to Saudi Arabia, Namib–1 and Patagonia dust. The uncertainty on XRF mass concentration takes into account loss correction uncertainties as detailed in Supplementary Methods. The uncertainty on LSM is the quadratic combination of LSM total mass concentration uncertainty (± 19%) and the standard deviation over the average period. The results of the linear fit are reported including also statistical indicators of the goodness of fit (correlation coefficient, R^2^, and reduced chi square, χ^2^_red_). Two linear fits are reported: Fit1 (blue) considering all data points, and Fit 2 (red) removing the three outlier points in blue from the dataset. The grey shaded area represents the range of Fit 1 uncertainty.

In order to have a further reference for the comparison of the mass concentration, we consider the one calculated from XRF elemental composition during CESAM experiments^64^. This estimate, based on mass determination of the main elements composing dust^72^, provides a robust evaluation, in excellent agreement with gravimetric measurements^19,64^. The comparison between LSM and XRF mass concentrations (Fig. 3) supports a very good agreement between the two techniques. The linear correlation between the two variables is excellent, as indicated by the correlation coefficient (R^2^ = 0.90; 0.98) and the reduced chi–square (χ^2^_red_ = 1.7; 0.3) (the two set of values are obtained by considering or not three outlier points in the dataset, see Fig. 3). The slope of 0.92 ± 0.12 (with the three outlier points included) and 0.69 ± 0.08 (outliers excluded) indicates an average 8 to 31% difference when fitting LSM and XRF data, which suggests a moderate but systematic underestimation of the mass concentration by LSM.

## Discussion

In this study, we perform a deconvolution analysis of the measured infrared extinction spectra of suspended natural dust aerosols to separate the contributions from its single constituent minerals and use these contributions to reconstruct the particles’ mineralogical composition and their spatio − temporal variability. Nineteen samples with contrasting mineralogy originated from worldwide sources are considered.

The results of the LSM inversion (mineral relative abundances, sample − to − sample variability) are in good agreement with both literature–based knowledge of dust mineralogical features and concurrent XRD measurements. Nevertheless, differences are found when comparing absolute values, in particular for quartz, calcite and feldspars between LSM and XRD. The XRD and LSM are looking at different matter properties, i.e., light–diffraction linked to crystalline structure in XRD versus chemical bonds leading to rotational and vibrational transitions in LWIR, and differences should be expected, as discussed in previous literature^48,49,57^. The tendencies in LSM–XRD differences in our study are in line with those found for sedimentary rocks investigations^57^.

The total mass concentration reconstructed from LSM is consistent with concurrent estimates from validated compositional − based analyses, which support the potential to recover both single minerals and total atmospheric dust mass loadings from measured LWIR optical signatures. However, despite an excellent linear correlation between LSM and XRF data, a potential systematic underestimation is identified from the analysis.

The LSM approach, i.e. its capacity to provide accurate mineral abundances, mineral relative proportions and total mass concentration, is dependent on the realism of the single mineral spectra in terms of the shape, intensity and position of absorption bands. Possible differences in size, morphology, and chemical state between reference and real minerals can affect the analysis. To this aim, and because of the well − known artefacts of the pellet technique^23,73^, it is important to have spectra for suspended minerals instead of compressed thin layer samples. Despite a number of reference spectra for suspended minerals is nowadays available for the main species composing dust, such as those used in the present analysis^74–77^, the ensemble remains limited. Also, we notice that most of reference spectra in the literature, including those used in this study, are obtained for submicron particles, while in nature most of these minerals extend up to several tenths of microns. In this regard, our analysis shows that the currently available library of reference minerals permits to well reproduce absorption bands, however the quality of the fit is not always perfect, and a non − negligible baseline contribution is needed to properly reproduce the spectra, especially at low wavenumbers. This could suggest that some mineral bands are not perfectly represented, and the fact that this occurs at low wavenumbers, where most of the interaction should come from coarse − sized particles, suggests a possible misrepresentation of submicron reference spectra compared to natural conditions. When considering the ensemble of reference spectra in the present analysis, it emerges that the dataset that more frequently contributes to LSM reconstruction and dominates the retrieved dust mass concentration is the one from Mogili et al.^77^, which is also the only one for which single mineral sizes extend up to super–micron diameters. The D_eff_ estimated for illite, kaolinite, montmorillonite, quartz and calcite in Mogili et al.^77^ is between 1.0 and 2.9 µm, which is at the lower bound but comparable to the values reported for our dust samples (Table 1), and significantly larger than the D_eff_ for the same minerals in additional references considered in our analysis, which instead remain below 0.6 µm^74,75^ (Supplementary Table 3). These results support the critical impact of the size distribution in reference spectra on the quality of the retrieval and reconstructed mineralogy. In agreement with previous studies^49^, our work suggests therefore that one limiting point for LSM application is the representativeness of reference spectra for single minerals, a dataset that should be consolidated and extended. Revising (or building up a new) database of single mineral mass extinction spectra that would include both the fine and the coarse mineral size distribution is needed. Spectra should be acquired on suspended particles and at high − resolution (2 cm^−1^ or better) in order to well record the characteristic features of the minerals which are key for their spectral speciation.

Despite limitations discussed in the previous paragraphs, the present analysis clearly proves that it is possible to retrieve the mineralogical composition of dust aerosols from worldwide sources and follow its changes during transport due to gravitational settling based on their LWIR extinction signature. Results from LSM deconvolution do not evidence any systematic trend in the observed dust mineralogy changes with time, with some of the samples showing modifications in clays partitioning and variations in feldspars content with time, and others not. In general terms, the fact that mineralogy does not evolve in a unique way for the different samples in our analysis suggests that the size − dependent composition of dust is possibly not the same between different sources, therefore leading to different temporal evolutions of the mineralogy due to gravitational settling. As a matter of fact, size − segregated compositional analyses of dust remains limited to a few case studies^20,32^ and low − level knowledge exists. Analyses conducted for Northern African dust in Morocco and Cape Verde^20,32^ show that the dust mineralogy varies with size, but the most important variations are below 0.5 and above 20 µm, while the composition is more uniform in between these limits. This kind of size − segregated mineralogy could explain why in some case we do not detect significant changes for changing size, knowing that our investigated size range is located mostly in the 0.5 − 20 µm limit.

The results of this study, show that the LSM analysis applied to infrared extinction spectra can be used to retrieve global − scale features of dust mineralogy, therefore pave the way to a new perspective of spectroscopic investigation of dust aerosols. In particular, the outcomes of this study are relevant for advancing the exploitation of past, current and next–generation hyperspectral satellite missions, as IASI and IASI − NG, in order to complement the retrievals in the UV or VSWIR, such as those from the new EMIT mission^51^. The increasing capabilities of current and future satellite instruments, in combination with new analyses approaches such as those presented in this paper, can allow to go further in the characterization of dust aerosols with remote sensing, thus providing new capabilities for quantifying their broad impacts on climate, air quality, and the environment. Combining UV, VSWIR and LWIR satellite measurements is expected to finally provide access to a comprehensive global scale size − resolved mineralogical information. Note that while the analysis provided here focuses on the mid–infrared region, the same considerations can be potentially extended to the far–infrared, and be of relevance also for the future FORUM satellite mission (Far-Infrared Outgoing Radiation Understanding and Monitoring), that will provide observations of atmospheric radiation up to 100 µm^78^, where spectral signatures of dust minerals can be found^28,79–81^. In addition, it is of relevance for ground–based infrared remote sensing observations, for example, but not limited to, those provided by NDACC (Network for the Detection of Atmospheric Composition Changes). Note that the lower limit of dust signal for which the LSM approach has been successfully applied in the present study is about 0.005–0.01 at 10 µm (as absorbance, a.u., that is 60–130 Mm^–1^ extinction coefficient accounting for the chamber optical pathlength). For 1 km deep uniform dust layer these extinctions would correspond to an aerosol optical depth of 0.06–0.1, a value that can be considered as the minimum to obtain the required signal–to–noise ratio to retrieve mineralogical information from a measured signature.

Using remote sensing observations to map dust mineralogy can provide new knowledge on the composition of dust laden air masses as they are emitted and transported across the globe, including areas not yet studied up to now because of difficult access (e.g. remote desert and oceanic areas), but also can provide insight on new potential source areas which are expected to progressively emerge in consequence of climate change − induced desertification. Indeed, because the LSM approach is sensitive to both crystalline, non − crystalline and amorphous compounds, it can reveal of high relevance for the study of high latitude and volcanic dust aerosols, composed of a significant amorphous and non − crystalline fraction^82,83^.

Global − scale mineralogical data can provide fundamental new input for improving Earth System Models performances to reproduce the dust atmospheric cycle. While soil mineralogical datasets are more and more integrated into model schemes^41,84^, including in perspectives those from the EMIT mission^51^, new global observational data are fundamental for either model assimilation and validation exercises, in particular for a better evaluation of dust climatic − relevant properties. Finally, retrieving mineralogical information on a large scale may also provide further understanding of the mechanisms behind dust variability and source identification, and can be also used for tracking of nutrients inputs to oceans and the ecosystems, fundamental for biogeochemical forcing attribution^13^.

As a concluding remark, the analysis presented in this study is based on laboratory extinction spectra measured on a spectral range and resolution comparable to those that can be detected from satellite hyperspectral sensors. It must be mentioned, however, that, compared to laboratory measurements, aerosol atmospheric observations from satellite are affected by the signal–to–noise ratio of current–generation infrared radiation measurements that can partly hide the aerosol signature. In the case of nadir–viewing instruments, possibly inhomogeneous vertical distribution of aerosols and the limited vertical sensitivity of the observations can also be a limiting factor in isolating the aerosol spectral signature. Additionally, for all types of real satellite observations, interference of infrared–absorbing gaseous species, clouds and the surface is also to be considered, from a full–radiative-transfer perspective. In this regard, studies focusing on the application of the LSM approach to real LWIR remote sensing observations are strongly needed.

## Methods

### Experimental data of infrared extinction spectra and physiochemical properties of natural suspended dust aerosols

Experimental dust data analysed in this work are obtained in the 4.2 m^3^ stainless‒steel CESAM chamber (French acronym for Experimental Multiphasic Atmospheric Simulation Chamber)^62^ and fully described in ^61,63,64,85^. The objective of the experiments is to investigate the spectral optical properties of mineral dust aerosols and their global − scale variability, going from UV − Vis to LWIR (0.37 − 16 µm, 625–27,027cm^–1^). To this aim a set of 19 natural soil samples with contrasted mineralogy and being representatives of the diversity of the surface composition at the global scale are selected based on concurrent information from a global soil mineralogy database^69^. The identified samples cover the four continents and both hemispheres and include Northern Africa (Algeria, Libya, Mauritania, Morocco, Tunisia), Sahel (Chad, Mali, Niger), Eastern Africa and Middle East (Ethiopia, Kuwait, Saudi Arabia), Eastern Asia (China), North America (Arizona), South America (Chile, Argentina), Southern Africa (Namibia), and Australia. Their list and origin are provided in Supplementary Table 1.

Mineral dust aerosols of these samples are generated by mechanical shaking of the natural parent soils, a procedure able to mimic the sandblasting of soils due to saltation during wind erosion as occurring in arid and semi − arid regions. This technique produces dust aerosol with realistic mineralogical composition and size distribution with respect to atmospheric conditions^61,63,85^. As discussed in Di Biagio et al.^61^, since the same protocol is applied for generating dust, differences in the size distribution and mass concentration for the various samples are related to differences in soil characteristics and its attitude to generate aerosols, in particular the coarse fraction. The dust aerosol produced by the mechanical shaking is injected into the CESAM volume and left in suspension for a time varying between 60 and 120 min while measuring the evolution of its physico‒chemical and spectral optical properties by a suite of instruments both measuring in situ and connected online to the chamber, as detailed in Supplementary Table 2. The only aging process occurring during the 120 min dust suspension in CESAM is gravitational settling, intended to reproduce the progressive loss of coarse particles during medium − to long − range transport of plumes in the real atmosphere. As discussed by Di Biagio et al.^61^, the dust number size distribution, measured by Scanning Mobility Particle Sizer (SMPS) and Optical Particle Counters (OPCs), extends up to tenths of µm at the peak of the injection and evolves rapidly during the experiments: particularly the coarse mode above 5 µm diameter rapidly decreases, and the observed dynamic well reproduces the size − selective changes observed in the field within 1 to 5 days of transport^39,70^. The mass concentration of dust aerosols in CESAM (derived from number size distribution assuming a density of 2.5 g cm^−3^^40,86^) reaches up to tenths of mg m^−3^ at the peak of the injection, in line with observations during dust storms close to source regions^20^, and reduces to values less than 1 to 2 mg m^−3^ after 120 min aging in CESAM (due to the combined effect of gravitational deposition and dilution due to instrumental sampling), comparable to what measured after several days of transport from African sources to the Mediterranean and the Atlantic^65,66^. These observations indicate therefore that a 2 h aging experiment in CESAM reproduces the temporal changes of size distribution and mass load of dust aerosols as observed in the real atmosphere from emission to medium − to long − range transport. As a proxy of the size distribution, the effective diameter (D_eff_) defined as ${{\varvec{D}}}_{{\varvec{e}}{\varvec{f}}{\varvec{f}}}=\frac{\sum_{{\varvec{i}}}{\varvec{\pi}}{{{\varvec{D}}}_{{\varvec{i}}}}^{3}{\varvec{d}}{{\varvec{N}}}_{{\varvec{i}}}}{\sum_{{\varvec{i}}}{\varvec{\pi}}{{{\varvec{D}}}_{{\varvec{i}}}}^{2}{\varvec{d}}{{\varvec{N}}}_{{\varvec{i}}}}$ is calculated for the different experiments. All chamber experiments are conducted at ambient laboratory temperature and dry conditions (relative humidity < 2%). Two experiments per soil type are usually conducted in order to test repeatability. The chamber is evacuated and kept at a pressure of 3∙10^−4^ hPa between each experiment, and manually cleaned between experiments with different soils to avoid carry − over contaminations. Background concentrations of aerosols in the chamber are less than 2.0 µg m^‒3^, which is about a factor of 1000 below operating conditions.

Concurrently with the size distribution and mass concentration, the evolution of the LWIR extinction spectrum of suspended dust aerosols is monitored online along the full duration of each experiment by means of an in situ Fourier Transform Infrared spectrometer (FTIR) interfaced with a multi − pass cell^62,87^ providing a total optical path length of (182.5 ± 0.5) m within the chamber. The dust extinction spectrum is retrieved from FTIR − measured absorbance between 625 and 5000 cm^−1^ (2 − 16 µm wavelength range) at 2 cm^−1^ resolution over 2 min intervals. For each experiment, the FTIR background spectrum is acquired immediately before dust injection in CESAM. Note that the FTIR spectra are degraded at 8 cm^−1^ resolution in the analysis to match reference spectra resolution (Supplementary Methods).

The bulk mineralogical composition of the dust aerosols in the size fraction < 10.6 µm diameter is obtained by X‒ray diffraction (XRD) on samples collected on filters over the whole duration of each experiment. Analysis of XRD spectra provided the mass proportion of clays, quartz, calcite, dolomite, gypsum, and feldspars in the aerosols. Additionally, X‒ray fluorescence (XRF) is performed on the filter samples to retrieve the dust elemental composition, then used to reconstruct the dust mass concentration during experiments^64,72^.

### LSM analysis of experimental extinction spectra

The LWIR extinction spectra measured in the CESAM chamber for natural dust aerosols mixtures are mathematically deconvoluted to represent them as the LSM of its single constituent minerals^48,56,57^. Under the hypothesis of external mixing, it is possible to write the spectral extinction of dust as a function of time as:

1 $${\mathrm{Ext}}_{\mathrm{Mod}}\left(\uplambda ,\mathrm{t}\right)=\sum_{\mathrm{i}=0}^{\mathrm{n}\_\mathrm{minerals}}{\mathrm{C}}_{\mathrm{i}}\left(t\right){\mathrm{Ext}}_{\mathrm{i}}\left(\uplambda \right)+{\cdot \mathrm{Ext}}_{\mathrm{b}}\left(\uplambda ,\mathrm{t}\right)$$

2 $$\mathrm{Residual}\left(\uplambda ,\mathrm{t}\right)={\mathrm{Ext}}_{\mathrm{Meas}}\left(\uplambda ,\mathrm{t}\right)-{\mathrm{Ext}}_{\mathrm{Mod}}\left(\uplambda ,\mathrm{t}\right)$$

where C_i_(t) are the coefficients representing the contribution of the mineral i to extinction spectra at the time t of the experiment, Ext_i_ (λ) is the reference extinction spectra for mineral i, Ext_b_(λ,t) is the offset term (polynomial baseline) of the equation, and Residual(λ,t) is the difference between measured, Ext_meas_(λ,t), and modelled, Ext_mod_(λ,t), spectra.

Using the estimated coefficients C_i_(t), the mass concentration (MC, µg m^−3^) contribution of each mineral to the dust mixture as a function of time can be calculated as:

3 $${MC}_{\mathrm{i}}\left(t\right)={\mathrm{C}}_{\mathrm{i}}\left(t\right){\cdot \mathrm{MC}}_{i,ref}\cdot \frac{{x}_{i,ref}}{{x}_{CESAM}}$$

where MC_i,ref_ is the mass concentration for the measured reference, and x_i,ref_ and x_CESAM_ are the pathlengths used for transmission spectroscopic measurements in the reference studies and in CESAM, respectively. The uncertainty on the derived MC_i_ is estimated to be 19% (90% confidence interval) (Supplementary Methods).

To determine the coefficients C_i_(t) and the Ext_b_(λ,t) in Eq. (1) an automatic least − squares fitting procedure is applied. The algorithm takes as input the measured extinction spectra and reference mineral spectra and provides the C_i_(t) and Ext_b_(λ,t) values that allows the best overlap between experimental and modelled data. The procedure calculates the baseline locally. Only a constraint for positive values is set to the calculation of C_i_(t) in the retrieval, despite in some isolated cases specific minerals have to be removed from the analysis since their inclusion provides unrealistic results. Calculations are performed at the same 2 min resolution of the FTIR spectra acquisition and restricted in the 740 − 1475 cm^−1^ range (6.8 to 13.5 µm), that is in the region where the most intense and spectrally varying dust signatures are found^23,28^. Note that above 1250 cm^−1^ (< 8 µm) the spectral signature of main dust minerals weakens considerably (Supplementary Fig. 2) with the only exception of carbonates and also that in this spectral region CESAM extinction spectra show additional noise due to possible residual contaminations by water vapor lines due to trace levels of H_2_O during experiments. Results are shown and discussed in the range 740 to 1250 cm^−1^ (8 − 13.5 µm, corresponding to the infrared atmospheric window) in the paper.

The Root Mean Square Error (RMSE) of the residuals, calculated as $\sqrt{\frac{\sum_{\lambda }{({Ext}_{mod}\left(\lambda \right)-{Ext}_{meas}\left(\lambda \right))}^{2}}{N}}$, with N the number of data points, is used to provide quality assessment of the LSM fitting. The Normalised Root Mean Square Error (NRMSE) is the ratio of the RMSE by the average Ext_mod_ (λ).

As two experiments per soil type were conducted, the LSM was repeated twice for each sample. The comparison of the results in the two cases demonstrates the repeatability of the whole procedure, both in retrieved minerals percent contributions and in temporal dynamics. However, we want to stress that the LSM analysis is conducted independently at each time step without any a priori information. This means that reproducibility of the results and the robustness of the spectral analysis is verified at each time step retrieval for each experiment.

### Library of reference extinction spectra

A library of reference extinction spectra for single minerals composing dust including silicates (phyllosilicates (mica, serpentine/kaolinite, smectite, chlorite, and talc/pyrophyllite groups) and tectosilicates (feldspar and silica groups) families), carbonates, and sulfates was constructed based on data available in the literature. For library constitution, we privileged datasets corresponding to measurements on suspended aerosol particles instead of compressed pellets, which are better representative of natural airborne aerosol samples and whose spectra are not affected by well − known artefacts including shift of absorption bands’ peak and modification of absorption band depth and profile^73^. Several studies fulfilled this requirement, allowing to collect data for major clay species (illite, kaolinite, montmorillonite), quartz, carbonates (calcite, dolomite), feldspars (albite, oligoclase), sulfates (anhydrite) and diatomaceous Earth (amorphous non − crystallized silica material)^74–77^. For other minerals, for which literature data on suspended particles are not available, we referred to classical pellet measurements, including references for five phyllosilicates (chamosite, chlorite, montmorillonite, serpentine, talc)^73^ and tectosilicates (orthoclase; Caltech mineral spectroscopy server). All reference mineral spectra were acquired for submicron − sized particles generally limiting to D < 2 µm, with the only exception of Mogili et al.^77^ that extended the range of measured diameters up to 7.5 µm. The spectral resolution was 8 cm^−1^ for the majority of the datasets. A summary of reference spectra used and conditions for their acquisition are provided in Supplementary Table 3 and detailed in Supplementary Methods. Supplementary Fig. 2 shows the reference spectra for different minerals used in this study. Note that for expected dominant minerals in dust (illite, kaolinite, montmorillonite, quartz, and calcite) various reference spectra from different literature studies are included in the library in order to evaluate the impact of different datasets in the process. Conversely, iron oxides are discarded, because of their relatively flat spectra which we tested to have a nil contribution to the deconvolution analysis of absorption peaks while potentially biasing the estimate of the baseline.

In addition to dust − composing minerals, we also included in the library the spectra of H_2_O and CO_2_, to account for possible contribution to the dust measured spectra. Also, ammonium sulfate aerosol spectra, presenting absorption bands at 7.0 and 8.9 µm^88^, is included as control non − dust specie to evaluate the deconvolution process. Control ammonium sulfate aerosol is obtained to have a negligible contribution (typically < 2%) to the total dust reconstructed mass for all samples.

### Supplementary Information

Supplementary Information.

## Data Availability

The simulation chamber experiments' data (LWIR spectra from FTIR, and total mass concentrations and effective diameters from size distribution) that support the findings of this study are available through the Database of Atmospheric Simulation Chamber Studies (DASCS) of the EUROCHAMP Data Centre (https://data.eurochamp.org/data-access/chamber-experiments/) with the identifiers listed in Supplementary Table 4. Reference spectra for single minerals are published in^74–77^. Data from Dorschner et al.^73^ are obtained from the Heidelberg—Jena—St.Petersburg—Database of Optical Constants (HJPDOC, https://www2.mpia-hd.mpg.de/HJPDOC/index.php; last access March 2023). Data from the Caltech Mineral Spectroscopy server are retrieved at http://minerals.gps.caltech.edu/files/Infrared_MIR/Minerals_From_JK/Index.htm; last access March 2023. The HITRAN (high-resolution transmission molecular absorption database) data are downloaded at https://hitran.org/about/ (last access March 2023). The XRD mineralogical data in Fig. 2 are from Di Biagio et al.^61^ (in their supporting information). The XRF mass concentration data in Fig. 3 are published in Caponi et al.^64^.

## Code availability

The Linear Spectral Mixing analysis was performed using ANIR POLWIN, a freely available routine developed at CEAM Foundation and distributed by the Eurochamp datacenter: https://data.eurochamp.org/anasoft/ (last access March 2023).
