# Supplementary material for: Infrared optical signature reveals the source–dependency and along–transport evolution of dust mineralogy as shown by laboratory study
Source: Sci Rep. 2023 Aug 15;13:13252. doi: 10.1038/s41598-023-39336-7 (PMC10427689; doi:10.1038/s41598-023-39336-7)
Supplement: Supplementary file 1 — Supplementary Information. [file 41598_2023_39336_MOESM1_ESM.pdf]

Supplementary information to "Infrared optical signature reveals the source–dependency and along–transport evolution of dust mineralogy as shown by laboratory study"

Claudia Di Biagio[1*], Jean-François Doussin[2], Mathieu Cazaunau[2], Edouard Pangui[2], Juan Cuesta[2], Pasquale Sellitto[2,3], Milagros Ródenas[4] and Paola Formenti[1]

[1] *Université Paris Cité and Univ Paris Est Creteil, CNRS, LISA, F-75013 Paris, France*
[2] *Univ Paris Est Creteil and Université Paris Cité, CNRS, LISA, F-94010 Créteil, France*
[3] *Istituto Nazionale di Geofisica e Vulcanologia, Osservartorio Etneo, Catania, Italy*
[4] *EUPHORE Labs., Fundación CEAM, 46980, Paterna, Valencia, Spain*

*Corresponding author. Email: claudia.dibiagio@lisa.ipsl.fr

Contents

1. Supplementary methods
   a. Detailed information on the library of reference spectra
      i) Reference tectosilicates and silica mineral spectra from Laskina et al.[1] – suspended aerosols
      ii) Reference phyllosilicate, tectosilicates, carbonates and sulfates mineral spectra from Mogili et al.[2] – suspended aerosols
      iii) Reference phyllosilicate, tectosilicates and carbonates mineral spectra from Hudson et al.[3,4] – suspended aerosols
      iv) Reference phyllosilicate mineral spectra from Dorschner et al.[5] – pellet data
      v) Reference tectosilicate mineral spectra from Caltech – pellet data
      vi) Additional reference spectra for gaseous compounds and ammonium sulfate aerosols
   b. Mass concentration from LSM: uncertainty calculations
   c. Details on calculations for comparison of LSM and XRF mass concentration data
2. Supplementary Tables
3. Supplementary Figures
4. Supplementary References

Supplementary Methods

a. Detailed information on the library of reference spectra

i. Reference tectosilicates and silica mineral spectra from Laskina et al.[1] – suspended aerosols

Simultaneous measurements of the aerosol infrared extinction spectra and number size distribution for three dust composing minerals (albite, oligoclase, diatomaceous earth) were obtained in the Multi-Analysis Aerosol Reactor System (MAARS[6,7]). Aerosol particles were generated by atomizing a solution of each mineral in ultrapure water (atomiser TSI Inc., Model 3076; ultrapure water Fisher Scientific, W7–4, Lot 111718). Particles were then dried to less than 5% relative humidity. Albite and oligoclase were purchased from WARD'S Natural Science Establishment, Inc. (Items 49 V 5851, Evje, Norway and 46 V 5803, Madawaska, Ontario, Canada, respectively) and diatomaceous earth compound was purchased from Alfa Aesar (Item 89381). Minerals (albite and oligoclase) were ground using a mortar and pestle in order to get fine powder samples.

The infrared extinction spectra of the single minerals were measured in the spectral range from 800 to 4000 cm^{-1} at 8 cm^{-1} resolution by means of a Thermo Nicolet, Nexus Model 670, FTIR spectrometer. Data were reported for the 800–1400 cm^{-1} spectral range in the publication. The pathlength of the FTIR within the measurement volume was reported to be (78 ± 7) cm [3]. The size distribution of the suspended mineral was retrieved by combining a scanning mobility particle sizer (SMPS, TSI, Inc., Model 3936) and an aerodynamic particle sizer (APS, TSI, Inc., Model 3321). The size of particles was limited to the accumulation mode range with diameters below 2.5 µm. In their study, Laskina et al.[1] report for the different samples a mass weighted mean diameter (D_w) of (586 ± 9), (491 ± 22), and (530 ± 21) nm for diatomaceous earth, albite and oligoclase, respectively. The shape factors retrieved from SMPS and APS data were 1.34 ± 0.02 for diatomaceous earth, 1.08 ± 0.02 for albite, and 1.05 ± 0.03 for oligoclase, indicating non spherical particles.

In our analysis we consider the FTIR extinction spectra (absorbance data, log10 base) from Laskina et al.[1] as reported in their Figures 1 and 2, and the number size distribution data as reported in their Figure S1 as a function of the volume equivalent diameter. The mass concentration of each mineral is retrieved from size data in our analysis assuming spherical shape and using the densities of 2.42 g cm^{-3} for diatomaceous earth, 2.62 g cm^{-3} for albite, and 2.65 g cm^{-3} for oligoclase, as reported in [1].

ii. Reference phyllosilicate, tectosilicates, carbonates and sulfates mineral spectra from Mogili et al.[2] – suspended aerosols

The extinction spectra for selected components of mineral dust aerosol were measured in a FEP–Teflon coated steel chamber (volume of 0.151 m^3). The mineral dust samples investigated in Mogili et al.[2] included illite, kaolinite and mormorillonite (Source Clay Repository), quartz (Strem Chemicals (>99.5%)), hematite (Sigma -Aldrich (>99.5%)), anhydrite (Alfa Aesar (>99.0%)), and calcite. For calcite, the extinction spectra were acquired for two different samples with different size distributions, one named calcite small (OMYA products (98.0%)) and another one named calcite large (EM science (>98.0%)). The samples were held under

vacuum for two hours (or were heated and pumped overnight in the case of montmorillonite for which water retention is higher) prior to the experiment to remove water. In the experimental procedure, the mineral sample was de–agglomerated and introduced into the chamber volume by using a pulsed solenoid valve coupled to an impactor plate. After a mixing time of about 10 min, the extinction spectra started to be collected continuously by means of a Mattson Infinity 60 AR FTIR spectrometer having a path length of (58.5 ± 0.5) cm in the chamber and collecting data in the range of 650–5000 cm^{-1} at 8 cm^{-1} resolution. Note that also UV VIS spectrometric measurements were performed in parallel with IR measurements during experiments to obtain mineral extinction at shorter wavelengths, and that the UV Vis spectrometer worked with a different path length. In order to account for the differences in path length between the two beams, the infrared extinction signal was scaled and normalized to a constant 100 cm path length. The FTIR measurements were performed at a time step of 53 s, corresponding to an average of 256 scans. All spectra were taken at room temperature (~296 K).

The size distribution for each mineral was not measured online but retrieved as the lognormal size distribution that allowed to best reproduce measured extinction spectra based on Mie theory calculations. The parameters of the lognormal function are reported in the Table 2 in the original paper and correspond to a modal diameter varying between 22 nm (quartz) to 357 nm (montmorillonite). Particles extending up to 7.5 µm diameters are reported in Mogili et al.[2], a value that is larger compared to the other available literature studies on suspended minerals that mostly measure submicron mineral aerosols[1,3,4]. As indicated in Supplementary Table 3, this results in supermicron D_w values varying between 1.7 µm for anhydrite to 4.2 µm for calcite large.

In our analysis we consider the FTIR extinction spectra (absorbance data, log10 base) from Mogili et al.[2] as reported in their Figures 2 to 4. The mass concentration of the mineral samples corresponding to the measured spectra is retrieved based on the number size distribution data recalculated from the lognormal parameters as in the original publication (Table 2 in Mogili et al.[2]), assuming spherical shape and using the densities of 2.8 g cm^{-3} for illite, 2.6 g cm^{-3} for kaolinite, and 2.35 g cm^{-3} for montmorillonite, 2.6 g cm^{-3} for quartz, 2.97 g cm^{-3} for anhydrite, and 2.71 g cm^{-3} for calcite, in line with densities reported in [3,4] for the same minerals. For mass concentration, and effective diameter calculations reported above, a size cut off of 7.5 µm is used.

iii. Reference phyllosilicate, tectosilicates and carbonates mineral spectra from Hudson et al.[3,4] – suspended aerosols

Single clay and non–clay mineral extinction spectra were retrieved in Hudson et al. [3,4] using the Multi-Analysis Aerosol Reactor System (MAARS[6,7]). The same experimental procedure was applied in the two studies. Considered clay minerals were illite (IMt-1), kaolinite (KGa-1, low defect) and montmorillonite (Cheto-SAz-1), all purchased from the Source Clays Repository, and non–clay minerals considered were quartz (Strem Chemicals, CAS-7631-86-9, Lot 143179-S), calcite (OMYAcarb UF), and dolomite (Spectrum, D1351, Lot QE0288).

Experiments for each sample consisted in suspending aerosol particles of the mineral sample and then in measuring simultaneously their infrared extinction spectra by means of a Fourier-

transform infrared (FTIR) spectrometer (Thermo Nicolet Nexus Model 670; liquid nitrogen cooled external MCT-A detector) and size distribution by combining an SMPS (TSI, Inc. Model 3936; DMA TSI, Inc. Model 3080, CPC TSI, Inc. Model 3025A), and an APS (TSI, Inc. Model 3321). The FTIR spectra were measured in the 800 to 4000 cm^{-1} spectral range at 8 cm^{-1} resolution by co–adding 256 scans. The aerosol samples were atomized from suspension of the clay material in ultrapure water (Optima water, Fisher Scientific, W7-4) using an atomiser (TSI, Inc. Model 3076). The illite sample was ground to get finer powder before use, whereas grounding was not applied to kaolinite and montmorillonite samples. The particles were dried to less than 15% relative humidity before entering the measuring volume by using a diffusion dryer (TSI, Inc. Model 3062).

The size distribution of the three aerosol suspensions was in the diameter range below 2 µm and the median diameter located at 153.6 nm (illite), 409.6 nm (kaolinite) and 208.8 nm (montmorillonite). For quartz and calcite, the size peaked at about 200 nm, while the peak was identified at about 50 nm for dolomite. The aerodynamic shape factors retrieved from combining mobility and aerodynamic number size measurements were 1.3, 1.1 and 1.1 for illite, kaolinite and montmorillonite, respectively, indicating non spherical particles. The experimental shape factors for quartz, calcite, and dolomite samples were determined to be 1.00, 1.05 and 0.097.

In our analysis we consider the FTIR extinction spectra (absorbance data, log10 base) as reported in Figures 3 to 5 and number size distribution data, reported as a function of the volume equivalent diameter, in Figure 1 in Hudson et al.[3,4]. The mass concentration of each mineral is retrieved from size data assuming spherical shape and using the densities of 2.8, 2.6 and 2.35 g cm^{-3} for illite, kaolinite and montmorillonite, respectively, and 2.6, 2.71, and 2.87 g cm^{-3} for quartz, calcite and dolomite, as reported in the original papers.

iv. Reference phyllosilicate mineral spectra from Dorschner et al.[5] *– pellet data*

In their study, Dorschner et al.[5], provided mass absorption coefficient (MAC) spectra (cm^2 g^{-1}) for five phyllosilicate minerals including chamosite (St. Brigitte, France), chlorite (Switzerland), montmorillonite (Com Zorlent, Romania), serpentine (Suarum, Norway) and talc (Oberderf, Austria). The collected ground material was grounded for 6 to 12 h to obtain submicronic grains, then they were pressed into KBr pellets. Electron microscopy analysis confirmed the irregular shape and size distribution below 1–2 µm for analysed grains, with number size peaking in the submicron fraction. Transmittance spectra were measured in the range 7.7 – 14.3 µm (1300 to 700 cm^{-1}) by a Zeiss UR 20 spectrometer. The spectral resolution of the dataset, originally at 2 cm^{-1}, is degraded to 8 cm^{-1} in the present analysis to homogenize with the other reference datasets.

In this study we consider the MAC spectra for the five minerals reported in the original paper in their Table 3. The mass concentration of the analyzed minerals to associate to each MAC spectra in our analysis is retrieved from the following calculations. First, we consider the mineral column density in the pellet as reported by the authors to be 3.18 10^{-4} g cm^{-2} for all minerals investigated, with the exception of talc for which it was 3.18 10^{-5} g cm^{-2}. The mass concentration in the pellet is retrieved assuming that the original disk is 1 mm thick and it has a surface of 1 cm^2, and by dividing the total column density by the thickness of the pellet, which

results in a mass concentration of 3.18 10^6 mg m^{-3} (3.18 10^5 mg m^{-3} for talc) in the pellet. Based on mass concentration data we estimate that the ratio between MAC spectra in cm^2 g^{-1} and absorbance spectra for Dorschner et al.[5] data is equal to a multiplicative factor of 7421. Given that MAC spectra are used in the deconvolution instead of absorbance spectra, as done for the other reference minerals, we account for this multiplicative factor in the mass concentration values for Dorschner et al. minerals when converting deconvolution coefficients $C_i(t)$ from LSM analysis into mineral mass concentrations. This results in a mass concentration of 2.30 10^{10} mg m^{-3} (2.30 10^9 mg m^{-3} for talc) to consider for this analysis.

v. Reference tectosilicate mineral spectra from Caltech – pellet data

The extinction spectrum for orthoclase feldspar is obtained from the Caltech Mineral Spectroscopy server (http://minerals.gps.caltech.edu/files/Infrared_MIR/Minerals_From_JK/Index.htm). Data correspond to KBr pellet spectroscopic measurements in the range 450 to 4400 cm^{-1} and were obtained by means of a Perkin Elmer FTIR Spectrometer. No information on the origin of the sample, size distribution and mass concentration of the material in the pellet is provided. In order to reconstruct a reasonable mass concentration to associate to the spectra we take as reference value a mineral column density of 3 10^{-4} g cm^{-2} in the pellet (a quite typical value in pellet spectroscopy corresponding to about 300 µg material diluted in 300 mg of KBr, also in line with the density reported in Dorschner et al.[5]), which corresponds to 3 10^6 mg m^{-3} mass concentration assuming a usual pellet of 1 mm thick and with a surface of 1 cm^2.

In our analysis we consider the FTIR transmission spectra (provided as % values from the Caltech Mineral Spectroscopy server) which we convert in absorbance assuming a log10 base.

vi. Additional reference spectra for gaseous compounds and ammonium sulfate aerosols

Extinction spectrum for gaseous compounds possibly present in the CESAM chamber during dust experiments and therefore visible in the FTIR signature have been added to the library. These include CO_2 and H_2O. For those species the spectra are retrieved from the HITRAN (high-resolution transmission molecular absorption database, https://hitran.org/about/), taking data at 300°C and 0.5 cm^{-1} resolution. The extinction spectrum of ammonium sulfate particles was acquired for suspended aerosols in CESAM and reported in Di Biagio et al.[8] has also been included as control species, as discussed in the main manuscript.

b. Mass concentration from LSM: uncertainty calculations

An error analysis is performed to constrain the uncertainty on the estimated single mineral and total mass concentration of dust obtained from the LSM procedure. The uncertainty in dust mass concentration from LSM arises from a combination of five uncertainties: 1/ uncertainty on CESAM absorbance spectra (±1% noise)[8] and 2/ CESAM optical path (±0.3%)[8]; 3/ uncertainty on reference mineral absorbance spectra (±1% noise) and 4/ optical path (±5%) (value at the average of those reported by[1–4]); 5/ uncertainty on the mass concentration of single reference minerals (±10%; estimated considering size distribution uncertainties and pellet column to volume concentration calculations detailed in the previous section). We do not take into account uncertainties on the coefficients C_i arising from the LSM procedure itself. Under

the hypothesis that the sources of uncertainty are independent, the 90% Confidence Interval (CI) from Gaussian standard distribution is calculated by adding in quadrature all mentioned uncertainties:

$$90\%CI=\pm 1.645\sqrt{\left((1\%)^2+(0.3\%)^2+(1\%)^2+(5\%)^2+(10\%)^2\right)}=\pm 19\% \qquad (1)$$

c. Details on calculations for comparison of LSM and XRF mass concentration data

The XRF mass concentration, i.e. the ratio of the mass deposited on the filter (µg) to the total volume of sampled air (m^3), is integrated over the dust sampling time on filter. In order to compare to XRF, the LSM mass concentration is averaged over the entire filter sampling period. Since LSM data refer to dust inside the CESAM volume, the mass concentration from XRF is corrected for particle losses along sampling lines from the chamber to the filter following the results in Di Biagio et al.[8]. Being L(D) the loss fraction (0–1) as a function of the particle diameter from the CESAM chamber to the sampling filter, the mass–weighted total loss integrated over the XRF filter sampling period (L_{XRF}) is calculated as:

$$L_{XRF}=\overline{ML(t)} \qquad (2)$$

$$ML(t)=1-\frac{\sum_i \frac{\pi}{6}D_i^3 dN(D_i,t)\rho\left(1-L(D_i)\right)}{\sum_i \frac{\pi}{6}D_i^3 dN(D_i,t)\rho} \qquad (3)$$

where $dN(D_i,t)$ is the number size distribution inside the CESAM chamber as retrieved from SMPS and OPC measurements and ρ is the dust density (set at 2.5 g cm^{-3} following literature[9,10]). The XRF mass concentration is then divided by (1 - L_{XRF}) to correct for particle losses along filter sampling lines. The L_{XRF} term varies between a minimum of 29% for Kuwait and Morocco to a maximum of 67% for the Arizona sample, in relation with the abundance of the coarse mode in the dust aerosols. As reported in Table 1 in the main manuscript, Kuwait and Morocco show amongst the lowest D_{eff} within the analysed samples and Arizona D_{eff} is amongst the highest values in the dataset. The uncertainty on the loss–corrected XRF mass concentration is estimated with the statistical error propagation formula taking into account the uncertainty on XRF mass concentration (±10% from comparison to gravimetric analysis in Caponi et al.[11]) and the uncertainty on L_{XRF} (between ±20 and 26%, accounting for a size–averaged 20% uncertainty for $L(D_i)$[8] and standard deviation of the average over sampling time in Eq. 2). The total uncertainty on the loss–corrected XRF mass concentration is in between 23 and 27%.

Supplementary Tables

Supplementary Table 1. List and origin of the dust natural samples analyzed in this study, as reported in Di Biagio et al.[8]. The collection coordinates indicate the location where the soil sample used for aerosol generation was originally collected.

Sample name	Collection Coordinates	Geographical zone	Country	Desert zone
Tunisia	33.02°N, 10.67°E	Northern Africa	Tunisia	Sahara desert (Maouna)
Morocco	31.97°N, 3.28°W	Northern Africa	Morocco	Sahara desert (east of Ksar Sahli)
Libya	27.01°N, 14.50°E	Northern Africa	Libya	Sahara desert (Sebha)
Algeria	23.95°N, 5.47°E	Northern Africa	Algeria	Sahara desert (Ti-n-Tekraouit)
Mauritania	20.16°N, 12.33°W	Northern Africa	Mauritania	Sahara desert (east of Aouinet Nchir)
Niger	13.52°N, 2.63°E	Sahel	Niger	Sahel (Banizoumbou)
Mali	17.62°N, 4.29°W	Sahel	Mali	Sahel (Dar el Beida)
Bodélé	17.23°N, 19.03°E	Sahel	Chad	Bodélé depression
Ethiopia	7.50°N, 38.65°E	Eastern Africa and the Middle East	Ethiopia	Lake Shala National Park
Saudi Arabia	27.49°N, 41.98°E	Eastern Africa and the Middle East	Saudi Arabia	Nefud desert
Kuwait	29.42°N, 47.69°E	Eastern Africa and the Middle East	Kuwait	Kuwaiti desert
Gobi	39.43°N, 105.67°E	Eastern Asia	China	Gobi desert
Taklimakan	41.83°N, 85.88°E	Eastern Asia	China	Taklimakan desert
Arizona	33.15 °N, 112.08°W	North America	Arizona	Sonoran desert
Atacama	23.72°S, 70.40°W	South America	Chile	Atacama desert
Patagonia	50.26°S, 71.50°W	South America	Argentina	Patagonian desert
Namib-1	21.24°S, 14.99°E	Southern Africa	Namibia	Namib desert (area between the Kuiseb and Ugab valleys)
Namib-2	19.0°S, 13.0°E	Southern Africa	Namibia	Namib desert (Damaraland, rocky area in north-western Namibia)
Australia	31.33°S, 140.33°E	Australia	Australia	Strzelecki Desert

Supplementary Table 2. List of instruments used during dust CESAM experiments, derived parameters, and uncertainty of relevance for the present analysis. For further details refer to Di Biagio et al.[8] and Caponi et al.[11].

**Instrument / technique**	**Parameter**	**Uncertainty**
In situ Fourier transform infrared Spectrometer (FTIR Bruker® Tensor 37™; liquid-nitrogen-cooled mercury cadmium telluride (MCT) detector, globar source), (2-16 μm)	Spectral absorbance	± 1% (noise), in some cases increasing up to 20% due to H_2O contamination at wavenumbers >1250 cm^{-1}<br>± 0.3% (optical path)++
Scanning mobility particle sizer (SMPS; TSI, DMA Model 3080, CPC Model 3772), used for geometrical diameter (D_g)<0.3 μm<br><br>SkyGrimm optical particle counter (Grimm Inc., model 1.129), used for 0.3<D_g<1.0 μm<br><br>WELAS optical particle counter (Palas, model 2000), used for D_g>1.0 μm	Number and mass size distribution	± 20-70%
	D_{eff} (effective diameter)	± 25%
X-ray diffraction (XRD, Panalytical model Empyrean diffractometer)	Total clays mass	± 14–100%
	Quartz mass	± 9%
	Feldspars mass	± 14% (orthose)<br>± 8% (albite)
	Calcite mass	± 11%
	Dolomite mass	± 10%
	Gypsum mass	± 18%
X-ray fluorescence (XRF, PW-2404 spectrometer by Panalytical)	Total dust mass	± 10%$$

++ Note that the evaluation of the optical path of the FTIR has been refined since Di Biagio et al.[8] where it was reported at 192 ± 4 m. It is now revised at 182.5 ± 0.5 m.

$$ From comparison against gravimetric measurements in Caponi et al.[11].

Supplementary Table 3. Information on reference extinction spectra used in this study. The table reports the mineral, its mineralogical family association, size distribution information on analysed samples, the mass concentration associated to the reference extinction spectrum, the path length of spectroscopic measurements, the resolution of acquired spectra, and the reference paper for the dataset. Data are reported as absorbance (using log10) for all reference studies with the exception of Dorschner et al.[5] for which data are MAC (mass absorption cross–section, $cm^2\ g^{-1}$). All measurements were acquired at 8 cm^{-1} resolution, with the exception of Dorschner et al.[5] acquired at 2 cm^{-1} resolution and the Caltech spectrum of orthoclase for which resolution is not reported. Details of the calculations and techniques are provided in Supplementary Methods. The uncertainty on the mass concentration of single reference minerals is evaluated as ±10% considering either size distribution uncertainties or pellet column to volume concentration calculations detailed in Supplementary Methods.

**Mineral**	**Mineral family**	**Size**	**Mass concentration (mg m^{-3}) (±10%)**	**Pathlength (m)**	**Reference**
Albite	Tectosilicates – feldspar group	D_w=0.491 µm, D_{eff}= 0.4 µm (D <2.5 µm)	4.3	0.78 ± 0.07	Laskina et al.[1]
Diatomaceous Earth or Diatomite	Amorphous silica material	D_w=0.586 µm, D_{eff}= 0.5 µm (D <2.5 µm)	3.7		
Oligoclase	Tectosilicates – feldspar group	D_w=0.530 µm, D_{eff}= 0.5 µm (D <2.5 µm)	5.0		
Illite	Phyllosilicates - mica group	D_{mod}=0.107 µm, σ=2.57 D_w=2.0 µm, D_{eff}= 1.0 µm (D <7.5 µm)	409.9	0.585 ± 0.005 (spectra adjusted to a path of 1.00 m)	Mogili et al.[2]
Kaolinite	Phyllosilicates - serpentine/kaolin group	D_{mod}=0.115 µm, σ=2.65 D_w=2.3 µm, D_{eff}= 1.1 µm (D <7.5 µm)	325.8		
Montmorillonite	Phyllosilicates - smectite group	D_{mod}=0.357 µm, σ=2.72 D_w=4.0 µm, D_{eff}=2.7 µm (D <7.5 µm)	379.3		
Quartz	Tectosilicates – silica group	D_{mod}=0.022 µm, σ=3.89 D_w=3.1 µm, D_{eff}= 1.4 µm (D <7.5 µm)	455.2		
Anhydrite	Sulfates	D_{mod}=0.100 µm, σ=2.50 D_w=1.7 µm, D_{eff}= 0.8 µm (D <7.5 µm)	363.0		
Calcite (small)	Carbonates	D_{mod}=0.052 µm, σ=2.68 D_w=1.4 µm, D_{eff}= 0.6 µm (D <7.5 µm)	756.2		
Calcite (large)	Carbonates	D_{mod}=0.271 µm, σ=3.06 D_w=4.2 µm, D_{eff}= 2.9 µm (D <7.5 µm)	160.7		

Illite	Phyllosilicates - mica group	D_{mod}=0.154 µm, σ=2.41<br>D_w=0.419 µm, D_{eff}= 0.3 µm<br>(D <2 µm)	41.9		
Kaolinite	Phyllosilicates - serpentine/kaolin group	D_{mod}=0.410 µm, σ=1.80<br>D_w=0.674 µm, D_{eff}= 0.6 µm<br>(D <2 µm)	42.8	0.78 ± 0.07	Hudson et al.[3]
Montmorillonite	Phyllosilicates - smectite group	D_{mod}=0.209 µm, σ=3.53<br>D_w=0.578 µm, D_{eff}= 0.4 µm<br>(D <2 µm)	28.1		
Quartz	Tectosilicates – silica group	D_{peak}~0.2 µm, D_w=0.615 µm<br>D_{eff}= 0.4 µm<br>(D <2 µm)	25.1		
Calcite	Carbonates	D_{peak}~0.2 µm, D_w=0.627 µm<br>D_{eff}= 0.4 µm<br>(D <2 µm)	158.9	0.78 ± 0.07	Hudson et al.[4]
Dolomite	Carbonates	D_{peak}~0.05 µm, D_w=0.546 µm<br>D_{eff}= 0.3 µm<br>(D <2 µm)	10.6		
Chamosite*	Phyllosilicate – Chlorite group	D <2 µm	$2.30\ 10^{10}$		
Chlorite*	Phyllosilicate – Chlorite group	D <2 µm	$2.30\ 10^{10}$		
Montmorillonite*	Phyllosilicates - smectite group	D <2 µm	$2.30\ 10^{10}$	0.001 (pellet thickness assumed to be 1 mm)	Dorschner et al.[5]
Serpentine*	Phyllosilicates - serpentine/kaolin group	D <2 µm	$2.30\ 10^{10}$		
Talc*	Phyllosilicates - talc/pyrophyllite group	D <2 µm	$2.30\ 10^{9}$		
Orthoclase*	Tectosilicates – fedspar group	–	$3.0\ 10^{6}$	0.001 (pellet thickness assumed to be 1 mm)	Caltech Mineral Spectroscopy server

D_{mod} = modal diameter and σ = width of the lognormal distribution retrieved from the original publication with the distribution expressed as $\frac{dN}{dlogD} = \frac{N_{tot}}{\sqrt{2\pi}ln(\sigma)} exp\left(-\frac{1}{2}\left(\frac{\ln\left(D/D_{mod}\right)}{\ln(\sigma)}\right)^2\right)$

D_{peak} = diameter at peak of the number distribution retrieved from original publication

D_w =mass weighted mean diameter retrieved from the original publication and defined as $\boldsymbol{D_w} = \frac{\sum_i D_i \frac{\pi D_i^3}{6} dN_i}{\sum_i \frac{\pi D_i^3}{6} dN_i}$

D_{eff} =effective diameter recalculated in the present work following the formulation $\boldsymbol{D_{eff}} = \frac{\sum_i \pi D_i^3 dN_i}{\sum_i \pi D_i^2 dN_i}$

** pellet data*

Supplementary Table 4. List of digital object identifier (doi) for the experimental data supporting the findings of this study. Those data are available through the Database of Atmospheric Simulation Chamber Studies (DASCS) of the EUROCHAMP Data Centre (https://data.eurochamp.org/data-access/chamber-experiments/). Each dataset can be cited as: "Di Biagio, C., Formenti, P., Doussin, J.-F., Cazaunau, M., & Pangui, E. (2023). *Atmospheric simulation chamber study: dust + None - Aerosol study - optical properties* [Data set]. AERIS." followed by the doi provided.

Sample name	Dataset doi reference in the EUROCHAMP Data Centre
Tunisia	https://doi.org/10.25326/E7ND-QB44
Morocco	https://doi.org/10.25326/PAVS-ZY18
Libya	https://doi.org/10.25326/CQ3X-BY88
Algeria	https://doi.org/10.25326/N01S-9P51
Mauritania	https://doi.org/10.25326/ERXH-PA09
Niger	https://doi.org/10.25326/FAJB-0944
Mali	https://doi.org/10.25326/KKZY-ET15
Bodélé	https://doi.org/10.25326/MMHQ-VW97
Ethiopia	https://doi.org/10.25326/XA75-T171
Saudi Arabia	https://doi.org/10.25326/4KY0-FZ61
Kuwait	https://doi.org/10.25326/4XAB-6595
Gobi	https://doi.org/10.25326/QEYS-Z786
Taklimakan	https://doi.org/10.25326/H2T5-TW21
Arizona	https://doi.org/10.25326/4PW7-PY36
Atacama	https://doi.org/10.25326/JJ00-ZN44
Patagonia	https://doi.org/10.25326/B2MX-P703
Namib-1	https://doi.org/10.25326/4A3X-5E25
Namib-2	https://doi.org/10.25326/M8M9-VT33
Australia	https://doi.org/10.25326/9CYT-W088

Supplementary Figures

Supplementary Figure 1. Illustration of Linear Spectral Mixing (LSM) analysis. Measured and modelled extinction spectra (expressed as absorbance in log10 convention) at the peak of the dust injection in the CESAM chamber and after 20 and 60 min aging time. The modelled spectra from LSM analysis is the sum of the single minerals contributions (Sum Minerals, plotted in red) and baseline offset term (Baseline, plotted in black dotted). The Residual (difference between measured and modelled signal) is also shown (plotted in grey). Examples of baseline and residuals are reported at the bottom of each plot in reference only to the spectra at 60 min. The horizontal black line represents the 0 value. Data are shown for all samples with exception of Tunisia, Australia and Kuwait for which the plots are already reported in Figure 1 in the main manuscript.

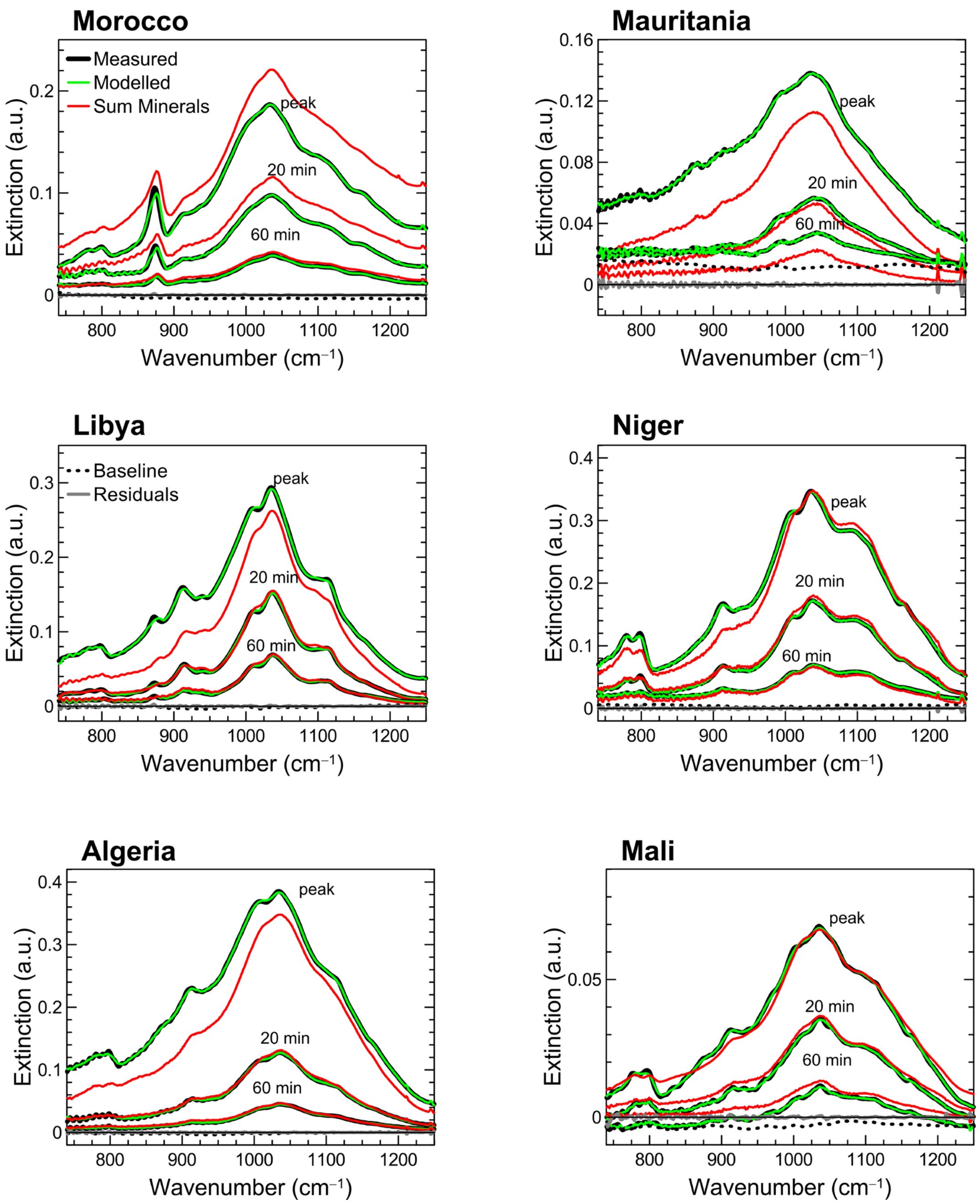

Supplementary Figure 1. Continued

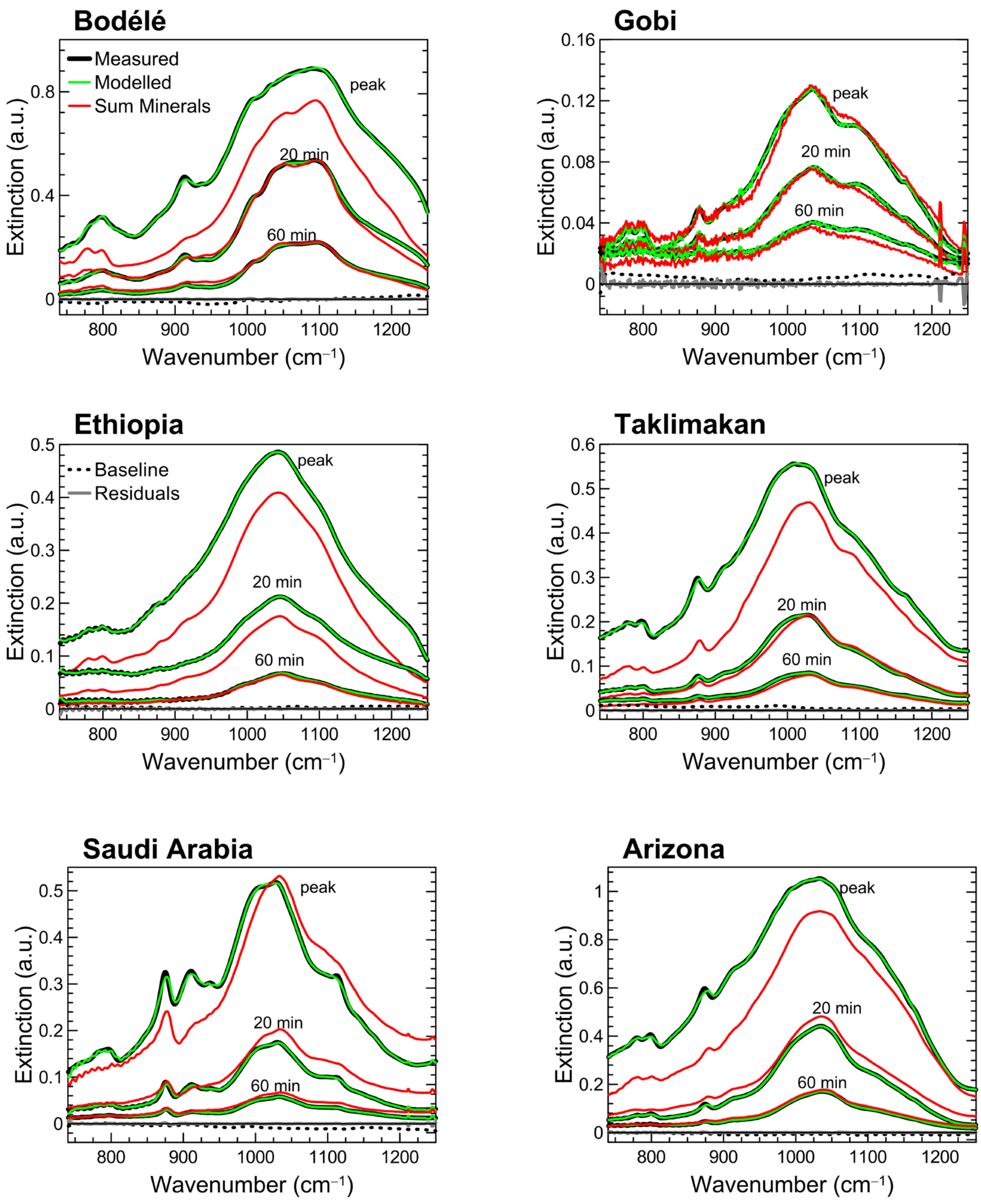

Supplementary Figure 1. Continued.

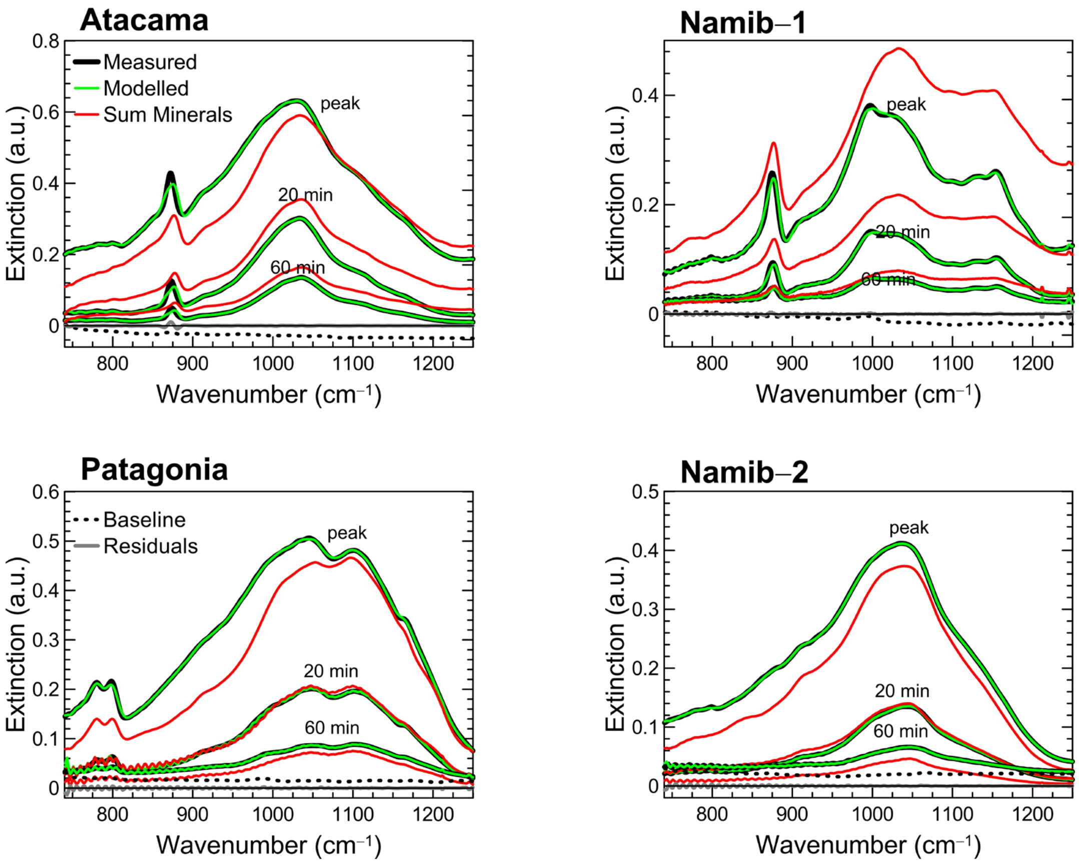

Supplementary Figure 2. Library of reference extinction spectra. Single mineral reference spectra (normalized by mass) for the different families considered, including phyllosilicates, tectosilicates, carbonates, sulfates, and amorphous silica. For all different reference studies, with the exception of Dorschner et al.[5], data are absorbance divided by total mass concentration, μg m^{-3}. For Dorschner et al.[5] data are MAC (mass absorption cross–section), cm^2 g^{-1}, divided by total mass concentration, μg m^{-3}. Data from Dorschner et al.[5] (chlorite, chamosite, montmorillonite, serpentine), Caltech (orthoclase), and Laskina et al.[1] (diatomaceous Earth) are plotted versus right side y–axis.

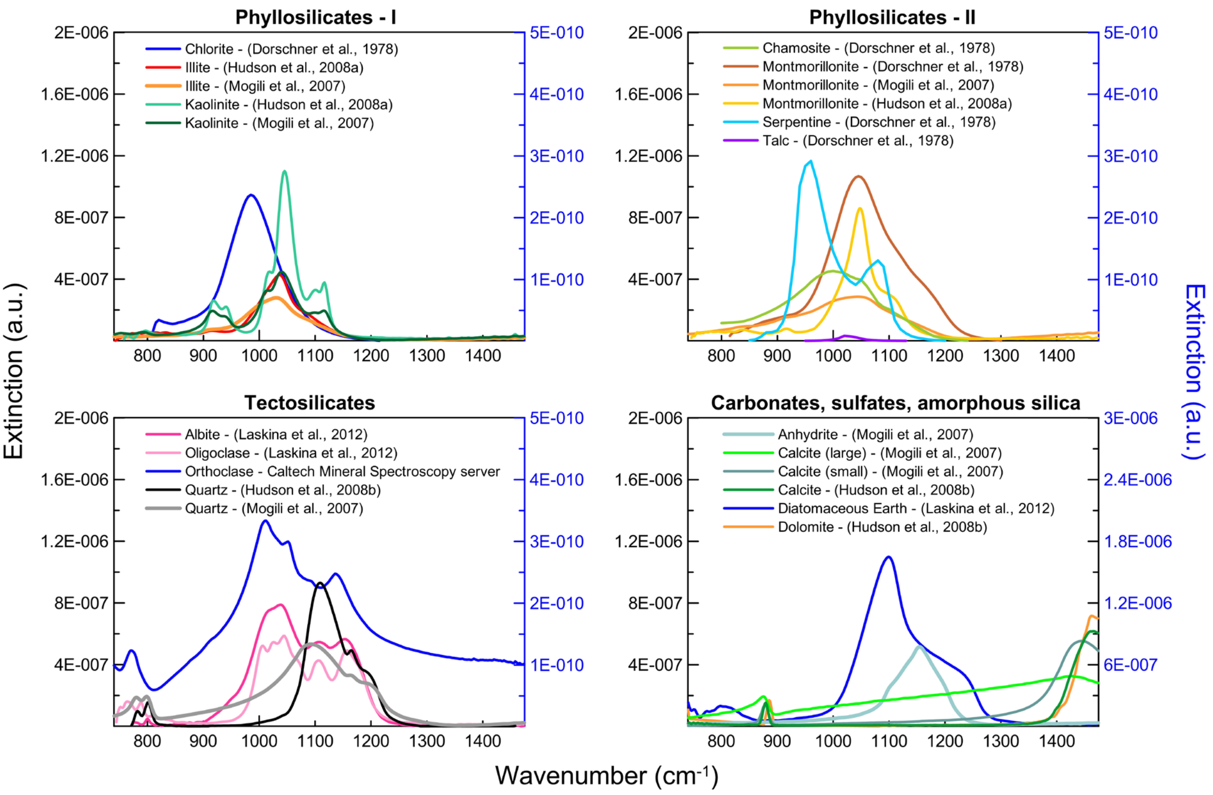

Supplementary Figure 3. Scatterplot of LSM versus XRD mineral mass percent abundances. Retrieved LSM percent content in mass of phyllosilicates, calcite, feldspars and quartz plotted against estimations from XRD. The solid line is the 1:1 line, the dotted and dashed lines represents ±10% and ±25% in mass percentage. Error bars represents LSM and XRD uncertainties. The tendencies in LSM–XRD differences in our dataset are in line with those found for sedimentary rocks investigations[12].

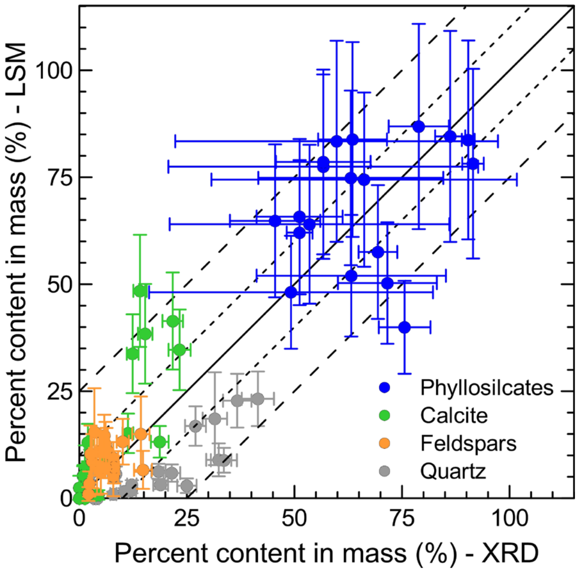

Supplementary References

1. Laskina, O., Young, M. A., Kleiber, P. D. & Grassian, V. H. Infrared extinction spectra of mineral dust aerosol: Single components and complex mixtures. *J. Geophys. Res. Atmospheres* **117**, (2012).
2. Mogili, P. K., Yang, K. H., Young, M. A., Kleiber, P. D. & Grassian, V. H. Environmental aerosol chamber studies of extinction spectra of mineral dust aerosol components: Broadband IR-UV extinction spectra. *J. Geophys. Res. Atmospheres* **112**, (2007).
3. Hudson, P. K., Gibson, E. R., Young, M. A., Kleiber, P. D. & Grassian, V. H. Coupled infrared extinction and size distribution measurements for several clay components of mineral dust aerosol. *J. Geophys. Res. Atmospheres* **113**, (2008).
4. Hudson, P. K., Young, M. A., Kleiber, P. D. & Grassian, V. H. Coupled infrared extinction spectra and size distribution measurements for several non-clay components of mineral dust aerosol (quartz, calcite, and dolomite). *Atmos. Environ.* **42**, 5991–5999 (2008).
5. Dorschner, J., Friedemann, C. & Gürtler, J. Laboratory spectra of phyllosilicates and the interstellar 10-micrometer absorption band. *Astron. Nachrichten* **299**, 269–282 (1978).
6. Gibson, E. R., Hudson, P. K. & Grassian, V. H. Physicochemical Properties of Nitrate Aerosols: Implications for the Atmosphere. *J. Phys. Chem. A* **110**, 11785–11799 (2006).
7. Hudson, P. K., Gibson, E. R., Young, M. A., Kleiber, P. D. & Grassian, V. H. A Newly Designed and Constructed Instrument for Coupled Infrared Extinction and Size Distribution Measurements of Aerosols. *Aerosol Sci. Technol.* **41**, 701–710 (2007).
8. Di Biagio, C. *et al.* Global scale variability of the mineral dust long-wave refractive index: a new dataset of in situ measurements for climate modeling and remote sensing. *Atmospheric Chem. Phys.* **17**, 1901–1929 (2017).
9. Reid, E. A. *et al.* Characterization of African dust transported to Puerto Rico by individual particle and size segregated bulk analysis. *J. Geophys. Res. Atmospheres* **108**, (2003).
10. Fratini, G., Ciccioli, P., Febo, A., Forgione, A. & Valentini, R. Size-segregated fluxes of mineral dust from a desert area of northern China by eddy covariance. *Atmospheric Chem. Phys.* **7**, 2839–2854 (2007).
11. Caponi, L. *et al.* Spectral- and size-resolved mass absorption efficiency of mineral dust aerosols in the shortwave spectrum: a simulation chamber study. *Atmospheric Chem. Phys.* **17**, 7175–7191 (2017).
12. Thorpe, M. T., Rogers, A. D., Bristow, T. F. & Pan, C. Quantitative compositional analysis of sedimentary materials using thermal emission spectroscopy: 1. Application to sedimentary rocks. *J. Geophys. Res. Planets* **120**, 1956–1983 (2015).
